# Dinucleosome specificity and allosteric switch of the ISW1a ATP-dependent chromatin remodeler in transcription regulation

**DOI:** 10.1038/s41467-020-19700-1

**Published:** 2020-11-20

**Authors:** Saurabh K. Bhardwaj, Solomon G. Hailu, Lola Olufemi, Sandipan Brahma, Soumyadipta Kundu, Swetansu K. Hota, Jim Persinger, Blaine Bartholomew

**Affiliations:** 1 The University of Texas MD Anderson Cancer Center, Department of Epigenetics & Molecular Carcinogenesis, Science Park, Smithville, TX 78957 USA; 2 Center for Cancer Epigenetics, MD Anderson Cancer Center, Houston, USA; 3 Worldwide Research and Development, Pfizer Inc, Houston, USA; 4 EpiCypher, Inc., Durham, USA; 5 National Institute of Neurological Disorders and Stroke, Bethesda, USA; 6 Division of Basic Sciences, Fred Hutchinson Cancer Center, Seattle, USA; 7 ZS Associates, Evanston, USA; 8 University of California-San Francisco, Gladstone Institutes, San Francisco, USA

**Keywords:** Enzyme mechanisms, Nucleosomes, Chromatin remodelling

## Abstract

Over the last 3 decades ATP-dependent chromatin remodelers have been thought to recognize chromatin at the level of single nucleosomes rather than higher-order organization of more than one nucleosome. We show the yeast ISW1a remodeler has such higher-order structural specificity, as manifested by large allosteric changes that activate the nucleosome remodeling and spacing activities of ISW1a when bound to dinucleosomes. Although the ATPase domain of Isw1 docks at the SHL2 position when ISW1a is bound to either mono- or di-nucleosomes, there are major differences in the interactions of the catalytic subunit Isw1 with the acidic pocket of nucleosomes and the accessory subunit Ioc3 with nucleosomal DNA. By mutational analysis and uncoupling of ISW1a’s dinucleosome specificity, we find that dinucleosome recognition is required by ISW1a for proper chromatin organization at promoters; as well as transcription regulation in combination with the histone acetyltransferase NuA4 and histone H2A.Z exchanger SWR1.

## Abstract (web summary)

Here the authors show that the preference of yeast chromatin remodeler ISW1a for dinucleosomes hinges on conformational changes that occur in the transition from binding mononucleosomes to dinucleosomes. These changes are critical for ISW1a organizing chromatin at promoters and regulating transcription in conjunction with other chromatin remodelers.

## Introduction

In *Saccharomyces cerevisiae*, there are two catalytic subunits called Isw1 and Isw2 that form three types of complexes. Isw1 assembled with the accessory subunit Ioc3 is called ISW1a or ISW1b when assembled with Ioc2 and Ioc4. ISW1a and ISW1b are localized, respectively, to regions immediately downstream of the NFR and into the coding region^1^. ISW1a represses transcription; whereas ISW1b assists in reassembling chromatin after the passage of the transcription complex and suppressing cryptic transcription in conjunction with CHD1^2–4^. Recruitment of ISW1b to coding regions is mediated by its Ioc4 accessory subunit and PWWP domain, a known “reader” of methylated lysine 36 of histone H3^2,5^. The factors directing ISW1a localization to the +1 and +2 nucleosomes are unknown, and unlike ISW1b, ISW1a with its Ioc3 and Isw1 subunits does not have any known histone modification reader domains. ISW1a had previously been proposed to bind dinucleosomes based on the X-ray crystal structure of truncated ISW1a lacking the ATPase domain bound to free DNA and protein-DNA crosslinking with two types of mononucleosomes^6^. These studies revealed that the Ioc3 subunit bound two distinct DNA segments and the HAND-SANT-SLIDE (HSS) domains of Isw1. A ISW1a-dinucleosome model was constructed based on these data, but has not been validated during the last nine years, even though it would be highly significant as thus far no other ATP-dependent chromatin remodeler is known to act specifically on dinucleosomes. ISW1a creates evenly spaced nucleosome arrays in vitro and is regulated in a linker-DNA length-dependent manner. If the dinucleosome model is valid for ISW1a then it would suggest a different mode for nucleosome spacing and linker-DNA dependent remodeling than currently proposed for protomers of Snf2h where Snf2h dimers bind to either side of mononucleosomes^7,8^.

Besides ISW1a in yeast, the NuA4 histone acetyltransferase and the SWR1 ATP-dependent chromatin remodeler that deposits H2A.Z are located at the 5′ end of genes and they genetically interact with each other^3,9,10^. It hasn’t been determined whether ISW1a or ISW1b is involved with these two remodelers and if it is involved with H2A.Z given NuA4 and SWR1 collectively promote deposition of H2A.Z^11–14^. A key structural difference between H2A.Z and H2A is the extended acidic patch of H2A.Z^15^. Mammalian ISWI complexes have been shown to bind the acidic patch of nucleosomes and to be required for efficient remodeling^16–18^. Expanding the acidic patch by incorporation of H2A.Z also stimulates mammalian ISWI nucleosome remodeling activity^19^. ISW1a interactions with the lateral histone surfaces of nucleosomes and “sensing” of the acidic pocket are not well understood, especially since in the ISWIa-dinucleosome model mentioned previously and in cryo-EM studies with Isw1 there is no evidence of Isw1 or Ioc3 contacting the acidic pocket or the histone octamer^6,20^.

We found large conformational changes between ISW1a interactions with mononucleosomes and dinucleosomes that impact nucleosome remodeling activity. Interactions with the acidic pocket of the histone octamer switches from primarily with Ioc3 in mononucleosomes to Isw1 in dinucleosomes. Also, Ioc3 binding to nucleosomal DNA at the dyad axis is lifted when binding dinucleosomes compared to mononucleosomes. These conformational changes are associated with ISW1a mobilizing dinucleosomes an order of magnitude more efficiently than mononucleosomes, even though the rate of ATP hydrolysis is equivalent for both substrates. We dissect these properties further by mutational analysis of Ioc3 and find its DNA binding domain is critical for these differences and show that it is required both in vitro and in vivo for ISW1a remodeling.

## Results

### ISW1a preferentially binds dinucleosomes rather than mononucleosomes

**Fig. 1:**
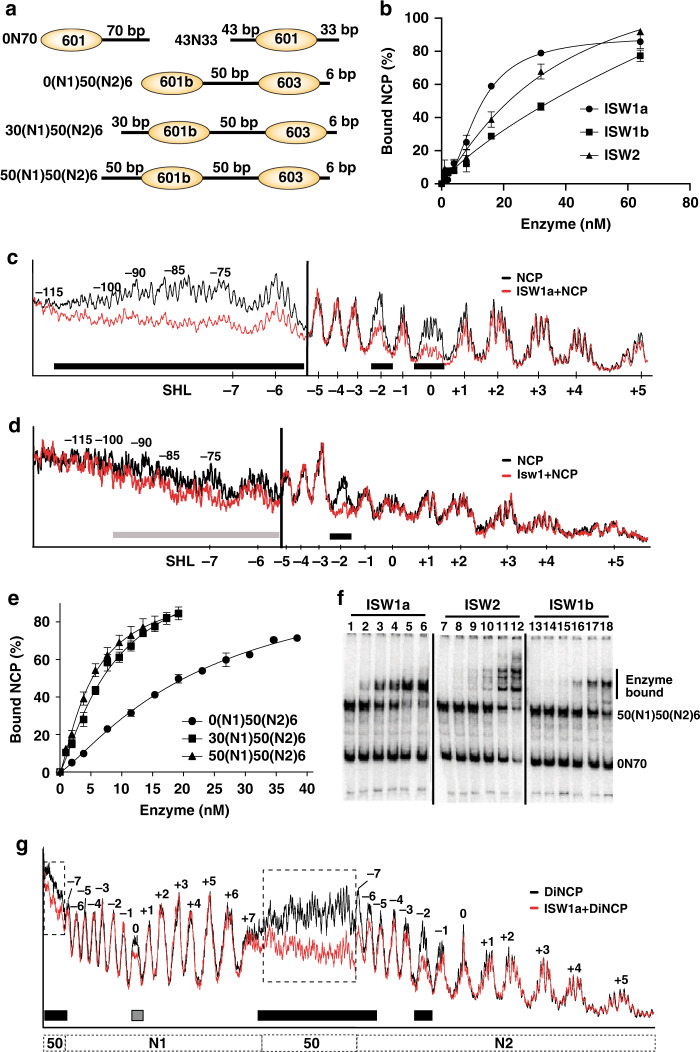
ISW1a has a higher affinity for dinucleosomes than mononucleosomes unlike ISW1b and ISW2. **a** Schematic shows the mono- and di-nucleosome substrates (light orange oval) used in this study. There are generally two types of mononucleosomes using the 601 Widom sequence with flanking DNA primarily on one side or approximately equivalent lengths of flanking DNA on both sides. There are three different dinucleosomal substrates that have in common 50 bp of DNA separating nucleosomes (NCP) assembled onto 601b and 603 nucleosome positioning sequences with 6 bp of DNA flanking the other side of the 603 nucleosome. The substrates varied based on the DNA length flanking the other side of the 601b nucleosome (0, 30, and 50 bp). **b** The affinities of ISW1a (closed circle), ISW1b (closed square) and ISW2 (closed triangles) for 0N70 mononucleosomes was measured by Electrophoretic Mobility Shift Assay (EMSA) using 20 nM nucleosomes and remodeler concentrations up to 60 nM. **c**, **d** The DNA interactions of (**c**) ISW1a and (**d**) Isw1 bound to 0N70 nucleosomes were mapped by DNA footprinting with hydroxyl radicals. The footprint with free nucleosomes is shown in black and with remodeler bound in red. Black and gray bars at the bottom of the trace marks the regions of strong and weak interactions. **e** The extent of 0(N1)50(N2)6 (closed circle), 30(N1)50(N2)6 (closed square) and 50(N1)50(N2)6 dinucleosomes (closed triangle) bound by wild-type ISW1a were plotted versus enzyme concentration. **f** The potential preference for binding 50(N1)50(N2)6 dinucleosomes versus 0N70 mononucleosomes with ISW1a, ISW2 or ISW1b was tested by EMSA using 15 nM each of mono- and di-nucleosomes and remodeler concentrations up to 20–25 nM. **g** DNA footprinting of ISW1a bound to 50(N1)50(N2)6 dinucleosomes was done similar to that described in (**c**, **d**). DNA footprinting experiments had a minimum of 2–3 replicates, all with similar results as those shown. All EMSA experiments were from *n* = 3 independent experiments and for those graphed the error bars are presented as mean values +/− SD. Source data are provided as a Source Data file.

We examined the nucleosomal substrate specificity of ISW1 with mononucleosomes containing 0 and 70 bp of DNA on either side of the 601 nucleosome positioning sequence (i.e., 0N70) and asymmetric dinucleosomes with 50 bp of linker DNA and flanking DNA of 0–50 and 6 bp length (Fig. 1a, see ref. ^21^). The affinity of ISW1a for 0N70 mononucleosomes was comparable to that of ISW1b and ISW2 as shown by EMSA (Fig. 1a and Supplementary Fig. 1a). Surprisingly, the affinity of Isw1 alone was similar to that of the ISW1a complex and DNA footprinting revealed Isw1 alone and ISW1a both bound to the SHL-2 and linker DNA positions (Fig. 1c, d and Supplementary Fig. 1b, c). ISW1a however bound tightly to the dyad axis and more extensively and stably to linker DNA unlike Isw1. ISW1a affinity for dinucleosomes varied based on the length of DNA flanking the N1 nucleosome with 50(N1)50(N2)6) dinucleosomes having a ~10 times higher affinity than without any flanking DNA and 2 times higher affinity than with 30 bp of flanking DNA, respectively a *K*_D_ of 5.29 nM compared to 45.9 and 10.2 nM (Table 1, Fig. 1e and Supplementary Fig. 1e). The affinity of ISW1a for 0N70 mononucleosomes (*K*_D_ = 30.3 nM) was intermediate between that for 0(N1)50(N2)6 and 30(N1)50(N2)6 ISW1a and was ~6 times lower than for 50(N1)50(N2)6 nucleosomes (Table 1). Unlike ISW1b or ISW2, the preference for dinucleosomes was unique to ISW1a as seen when titrating these remodelers into an equimolar mix of 0N70 mononucleosomes and 50(N1)50(N2)6 dinucleosomes. Subsaturating amounts of ISW1a preferentially bound dinucleosomes over mononucleosomes as observed by EMSA; whereas ISW1b and ISW2 bound dinucleosomes and mononucleosomes equally well (Fig. 1e). ISW1a bound dinucleosomes as a monomer consisting of one subunit each of Isw1 and Ioc3 rather than as a protomer as shown by the Hill coefficient ranging from 1.17 to 1.43 and only one bound species being detected at the different ISW1a concentrations (Table 1 and ref. ^22^). DNA footprinting also suggests that ISW1a bound as a monomer given the SHL-2 and SHL-6/-7 positions of N2 nucleosomes are bound, while ISW1a did not engage N1 nucleosomes (Fig. 1g).

**Table 1:** Affinities of wild-type and mutant ISW1a and Isw1 for mononucleosomes and dinucleosomes.

ISW1a					ΔcHLB		ISW1a	Isw1	DcHLB
Nucleosome	0N_1_50N_2_6	30N_1_50N_2_6	50N_1_50N_2_6	0N_1_50N_2_6	30N_1_50N_2_6	50N_1_50N_2_6	0N70	0N70	0N70
*K*_D_ (nM)	45.9	10.2	5.29	34.5	28	12.5	30.3	21.9	32.5
95% CI	29.0–134	7.81–15.9	4.62–6.39	21.1–193	20.7–52.8	10.4–16.4	19.4–42.2	10.9–37.1	15.2-53.7
*H*	1.17	1.19	1.43	1.2	1.3	1.25	1.35	n.d.	n.d.
95% CI	0.921–1.45	1.01–1.39	1.22–1.67	0.812–1.66	1.01–1.64	1.02–1.50	0.895–1.96	n.d.	n.d.
*R*^2^	0.997	0.998	0.998	0.992	0.996	0.996	0.968	0.922	0.958

### ISW1a preferentially mobilizes dinucleosomes over mononucleosomes

**Fig. 2:**
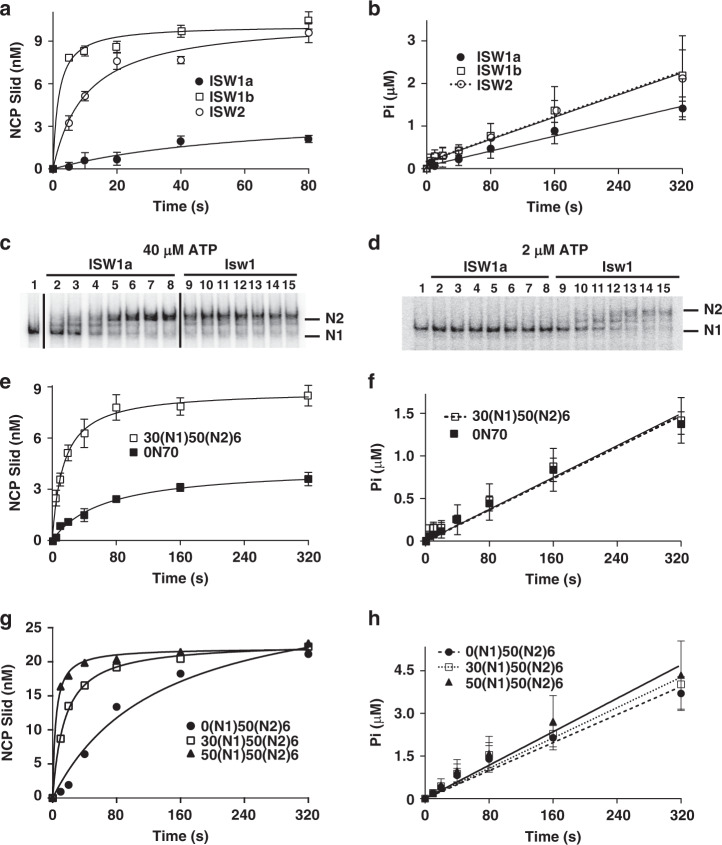
ISW1a preferentially remodels dinucleosomes over mononucleosomes. **a**, **e** and **g** The rate ISW1a mobilizes various nucleosomal substrates was determined by EMSA for one round of remodeling with remodeler in excess to nucleosomal substrate. The efficiency of ISW1a remodeling was compared to either (**a**) ISW1b and ISW2 with 0N70 mononucleosomes or (**e**, **g**) ISW1a with mono- and dinucleosomes. The initial rates of remodeling by ISW1a, ISW2 and ISW1b were respectively 0.060 ± 0.033, 0.77 ± 0.084 and 1.2 ± 0.12 nM s^−1^ with 40 μM ATP, 20 nM 0N70 mononucleosomes, and 80 nM ISW1a, ISW1b or ISW2. In (**e**) and (**g**) the conditions were the same, except for 16 μM ATP in (**e**) and 2 μM ATP and 800 μM γ-S-ATP in (**g**). In (**e**) initial rates of 0N70 and 30(N1)50(N2)6 remodeling by ISW1a were estimated to be respectively 0.063 ± 0.018 and 0.51 ± 0.027 nM s^−1^. The initial rate of remodeling by ISW1a for 0(N1)50(N2)6, 30(N1)50(N2)6 and 50(N1)50(N2)6 in (**g**) were estimated to be respectively 0.23 ± 0.099, 1.2 ± 0.011 and 2.8 ± 0.11 nM s^−1^. **b**, **f** and **h** The rates of ATP hydrolysis for the remodeling conditions described above were determined using γ-^32^P-ATP and thin-layer chromatography. **b** The amount of inorganic phosphate (Pi) generated was plotted versus time and the observed rates of ATP hydrolysis by ISW2, ISW1a and ISW1b with 0N70 mononucleosomes were respectively 6.5 ± 0.35, 4.4 ± 0.16 and 6.6 ± 0.36 nM s^−1^. **f** The rates of ATP hydrolysis of ISW1a with 0N70 and 30(N1)50(N2)6 mono- and di-nucleosome were respectively 4.7 ± 0.19 and 4.6 ± 0.21 nM s^−1^. In (**h**) 40 µM was used instead of the ATP/γ-S-ATP mix and the rates of ATP hydrolysis for ISW1a with 0(N1)50(N2)6, 30(N1)50(N2)6 and 50(N1)50(N2)6 were respectively 12 ± 0.70, 13 ± 0.75, 15 ± 0.88 nM s^−1^. **c**, **d** The rates of ISW1a (lanes 1–8) and Isw1 (lanes 9–16) remodeling 0N70 nucleosome was determined as described in (**a**) with the ATP concentration at (**c**) 40 or (**d**) 2 µM ATP as indicated. Reaction times were 0, 10, 20, 40, 80, 160, 320, and 640 s. Nucleosome remodeling and ATPase assays were all done in n = 3 independent experiments and error bars are presented as mean values +/− SD. Source data are provided as a Source Data file.

The efficiency of ISW1a remodeling mono- and di-nucleosomes was compared by saturating nucleosomes with ISW1a and observing the rate of remodeling by EMSA for a single round of remodeling. ISW1a remodeled 0N70 mononucleosomes 13- and 20-times slower than ISW2 and ISW1b without any significant difference in ATP hydrolysis (Fig. 2a, b and Supplementary Fig. 2a–c). The remodeling efficiency of ISW1a compared to Isw1 alone was also 12-times slower without a noticeable difference in ATP hydrolysis (Fig. 2c, d and Supplementary Fig. 2d). The inefficient remodeling of ISW1a was reversed when remodeling dinucleosomes with 30(N1)50(N2)6 dinucleosomes remodeled 8-times faster than 0N70 nucleosomes (Fig. 2e, f and Supplementary Fig. 2e, f). DNA flanking N1 nucleosomes is critical for efficient dinucleosome remodeling as fully bound dinucleosomes lacking flanking DNA on the N1 side remodeled 5.4 and 10 times less efficiently respectively than 30(N1)50(N2)6 and 50(N1)50(N2)6 dinucleosomes without differences in ATP hydrolysis (Fig. 2g and Supplementary Fig. 2g–i). DNA footprinting of 0N70 mononucleosomes and 50(N1)50(N2)6 suggest differences in remodeling could be due to the lack of interactions at the dyad and not to changes in binding of the ATPase domain.

### ISW1a only moves DNA in a backtracking motion when remodeling mononucleosomes

**Fig. 3:**
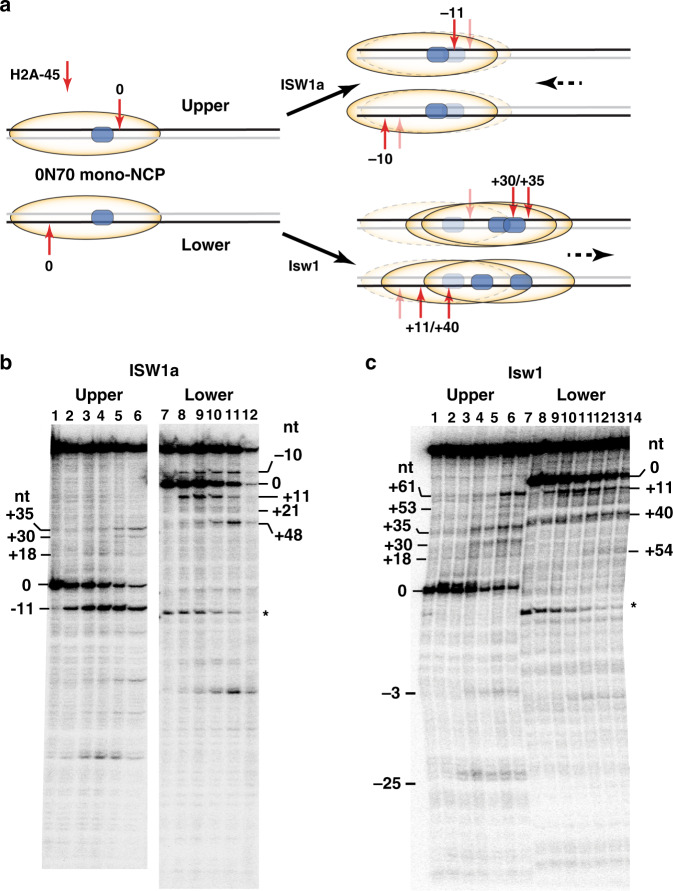
The Ioc3 subunit reduces nucleosome mobilization by promoting backtracking of Isw1. **a** DNA movement during remodeling of 0N70 nucleosomes was tracked by mapping DNA contacts with histones by modifying residue 45 of histone H2A (red arrow) and photocrosslinking DNA as depicted. Two sides of the dyad axis are tracked by labeling either the upper or lower strands with ^32^P at the 5′ end of DNA. The labeled strand is shown in black and the unlabeled strand in gray. Shown in the schematic is the nucleosome movement on DNA when remodeled either by ISW1a or Isw1 and the starting contact point is referred to as zero. The bound location of the ATPase domain is shown in blue. The remodeled nucleosome positions are shown along with an outline of the original nucleosome for reference. **b**, **c** The phosphorimages are shown for (**b**) ISW1a and (**c**) Isw1 samples analyzed on a 6% polyacrylamide with 8 M urea. Nucleosomes with the upper (lanes 1–6) or lower (lanes 7–12) DNA strands labeled were separately analyzed and experiments were done with *n* = 3 independent replicates.

DNA movement inside of mononucleosomes during remodeling were tracked by replacing residue 45 of histone H2A with cysteine, coupling it to a photoreactive probe, crosslinking and cleaving the proximal DNA. Cleavage occurs 37–39 bp from the dyad axis and is DNA strand-specific, such that DNA movement close to the ATPase domain (upper-strand) or on the other side of the dyad axis (lower-strand) was followed (Fig. 3a). When ISW1a remodeled 0N70 nucleosomes, DNA closest to the ATPase domain moved 11 bp in the opposite direction (i.e., backtracking) than for nucleosomes moving onto extranucleosomal DNA (Fig. 3a, b, −11 nt in lanes 1–6), while less efficient movement was observed in the other direction (see nts +18, +30 and +38). Similar backtracking motion was observed on the other side of the dyad axis with the lower-strand of DNA labeled, but was much less pronounced than the forward movement of DNA (Fig. 3b, lanes 7–12 compare nt −10 versus nts +11, +21 and +48). The differences in DNA movement dependent on proximity to the ATPase domain suggests a block at the dyad axis could interfere with DNA passage and reverse the direction of DNA movement near the ATPase domain. Isw1 alone does not cause backtracking of DNA and DNA moves more efficiently in the forward direction than ISW1a (Fig. 3a, c, lanes 1–6 and lanes 7–12).

### Ioc3 subunit binds to the dyad axis and potentially blocks DNA passage through nucleosomes

**Fig. 4:**
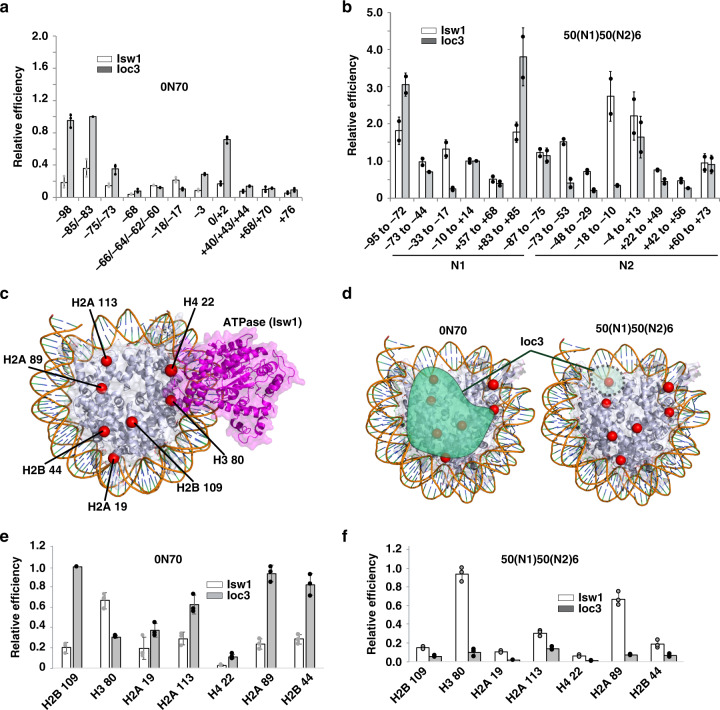
Allosteric changes in Isw1 and Ioc3 interactions with dinucleosomes revealed by site-directed DNA and histone crosslinking. **a**, **b** The nucleosomal and extranucleosomal DNA binding sites of Isw1 and Ioc3 were mapped by DNA crosslinking with (**a**) 0N70 mononucleosomes and (**b**) 50(N1)50(N2)6 dinucleosomes. Numbering refers to the number of nucleotides from the dyad axis (0) where photoreactive residues were incorporated. Open bars are for Isw1 and closed bars for Ioc3. **c** Site-directed histone crosslinking at total of 7 sites (red spheres) was used to map the interactions of Isw1 and Ioc3 with the lateral surface(s) of mono- and di-nucleosomes. The location of the Isw1 ATPase domain shown in magenta is from a recent cryo-EM study (PDB 6IRO^20^). **d** Summary of where Ioc3 binds nucleosomes when ISW1a is bound to either 0N70 mononucleosomes or 50(N1)50(N2)6 dinucleosome is shown (highlighted in teal). The different sites being probed inside of nucleosomes are depicted as in (**c**). **e**, **f** The extent of Ioc3 and Isw1 crosslinked to the indicated modified histone residue is shown for (**e**) 0N70 and (**f**) 50(N1)50(N2)6 dinucleosome. The open and closed bars are respectively Isw1 and Ioc3. Nucleosomes were fully bound and had 20 nM of mono- or dinucleosomes with respectively 25 or 40 nM ISW1a for mono- and dinucleosome reactions. DNA (orange) and histones (gray) are depicted. All histone crosslinking experiments were from *n* = 3 independent experiments and error bars are presented as mean values +/− SD. Source data are provided as a Source Data file.

Site-specific DNA photocrosslinking showed Ioc3 was localized primarily to the dyad axis of mononucleosomes and DNA 10–25 nts from the edge of the nucleosome (Fig. 4a, see nts 0/+2, −85/−83 and −98 and Supplementary Figs. 3, 4 and 5a). Ioc3 was previously thought to bind only to extranucleosomal DNA and not at the dyad axis, which could be due to the lack of the ATPase domain in these earlier studies^6^. DNA crosslinking revealed Isw1 is associated at the SHL-2 position and Ioc3 doesn’t bind at the dyad axis of N2 nucleosomes (Fig. 4a and Supplementary Fig. 5b). These data suggest that dinucleosomes are remodeled better than mononucleosomes because Ioc3 binds DNA at the dyad axis of only mononucleosomes which in turn blocks DNA passage.

### Isw1 primarily engages the acid pocket and lateral surface of nucleosomes upon binding to dinucleosomes but not mononucleosomes

Seven sites in the histone octamer were independently coupled to a photoreactive reporter in 0N70 and 50(N1)50(N2)6 dinucleosomes to probe the interactions of Ioc3 and Isw1 with the lateral faces of nucleosomes^23–25^. Ioc3 associates with the lateral face of mononucleosomes as shown by crosslinking to five out of seven positions and was the dominant subunit bound at the acidic pocket of nucleosomes and Isw1 crosslinked to residue 80 of histone H3, close to the SHL-2 position (Fig. 4c–e and Supplementary Fig. 5c, d; residues H2A-89, H2B-109, and H2A-113). Extensive Ioc3 interactions with the lateral face of mononucleosomes is inconsistent with previous mononucleosome data used for modeling ISW1a bound to dinucleosomes^6^. The histone crosslinking pattern of ISW1a with dinucleosomes had Isw1 crosslinking to the lateral surfaces and acidic pocket of dinucleosomes rather than Ioc3 (Fig. 4e and Supplementary Fig. 5e, see H380, H2A89, and H2A113). Similar to our data, binding of the mammalian homolog of Isw1, Snf2h, to the acidic pocket is required to stimulate the remodeling activity of the ISWI complex by relieving auto-inhibition^16–18^. Two changes in ISW1a interactions with nucleosomes are required to deactivate ISW1a when remodeling mononucleosomes that are switched with dinucleosomes.

### The HLB domain of Ioc3 is required for proper recruitment of ISW1a to dinucleosomes, but not to mononucleosomes

**Fig. 5:**
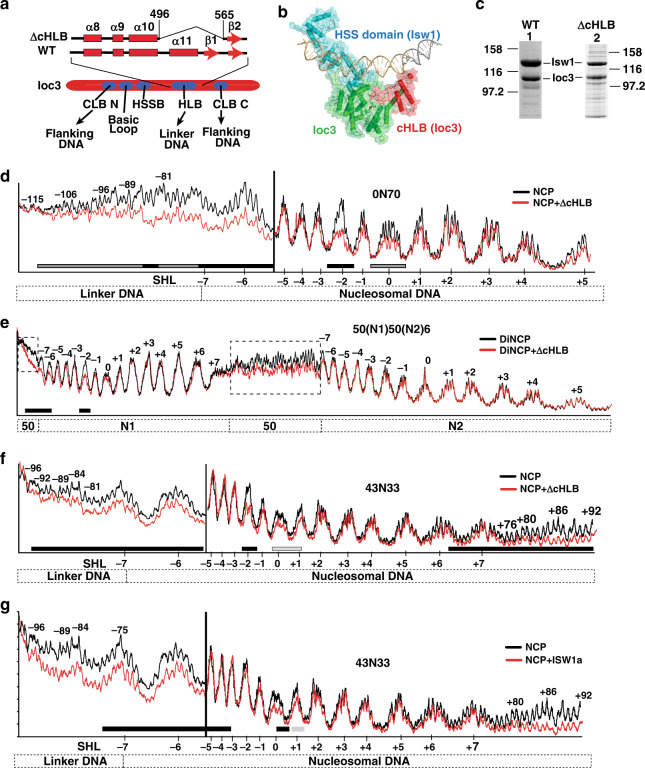
Binding of the HLB domain of Ioc3 to extranucleosomal DNA is required for ISW1a to bind dinucleosomes. **a** The HLB domain of Ioc3 (red) was partially truncated as depicted to disrupt its binding to extranucleosomal or linker DNA. Ioc3 domains are shown in blue. **b** The crystal structure of truncated ISW1a with free DNA is shown and the region of HLB that was deleted in red^6^. The HSS domain of Isw1 (cyan) and Ioc3 (green) are shown. **c** Wild-type and mutant ISW1a with the C-terminal end of HLB deleted (ΔcHLB) were analyzed by SDS-PAGE and stained with Coomassie blue. **d**–**g** The interactions of ΔcHLB ISW1a with (**d**) 0N70, (**e**) 50(N1)50(N2)6 and (**f**) 43N33 nucleosomes or (**g**) wild-type ISW1a with 43N33 nucleosomes were analyzed by DNA footprinting with hydroxyl radicals. Black traces are nucleosome alone and red are the nucleosomes fully bound by wild-type or mutant ISW1a. DNA footprinting experiments had a minimum of 2–3 replicates with similar results as those depicted. Source data are provided as a Source Data file.

We deleted the α11 helix of the helical-linker DNA binding (HLB) domain in Ioc3 as well as the short, flanking β1 beta sheet (∆cHLB) to disrupt HLB function and found by SDS-PAGE that ISW1a complex integrity was retained (Fig. 5a–c). Many of the normal contacts of ISW1a with mononucleosomes were maintained with binding to the SHL2 position and linker DNA with ISW1a-∆cHLB (compare Fig. 5d to Fig. 1c). Interactions with the more distal linker DNA spanning from nts −96 to −115 were however lost or reduced, consistent with that expected for loss of the HLB domain binding DNA^6^. ISW1a interactions with dinucleosomes were however dramatically altered upon truncation of the HLB domain with ISW1a binding to N2 nucleosomes lost (see SHL-2 and SHL-6/-7) and no extensive binding to the linker DNA connecting N1 and N2 nucleosomes (compare Fig. 5e to Fig. 1g). Instead, the ∆cHLB complex switched binding to the N1 nucleosome as evident by gain of protection at the SHL-2 and SHL-6/-7 of N1 and 50 bp of DNA flanking the N1 nucleosome, and resembles the ∆cHLB complex bound to 43N33 mononucleosomes (compare Fig. 5e, f). The affinity of ∆cHLB ISW1a for 0N70 mononucleosomes was nearly the same as that for wild-type ISW1a, (Supplementary Fig. 1b–c and Table 1); however was 2.4–2.7 times less for 30(N1)50(N2)6 and 50(N1)50(N2)6 dinucleosomes than wild type, consistent with the ∆cHLB complex binding only N1 of the dinucleosome (Supplementary Fig. 6 and Table 1). These data suggest that the inability of ISW1a to bind to the distal linker DNA, as seen with mononucleosomes, blocks the appropriate allosteric switch required for proper docking of ISW1a onto dinucleosomes.

### Proper recruitment of ISW1a to dinucleosomes and DNA sensing by the HLB domain is required for nucleosome spacing by ISW1a

**Fig. 6:**
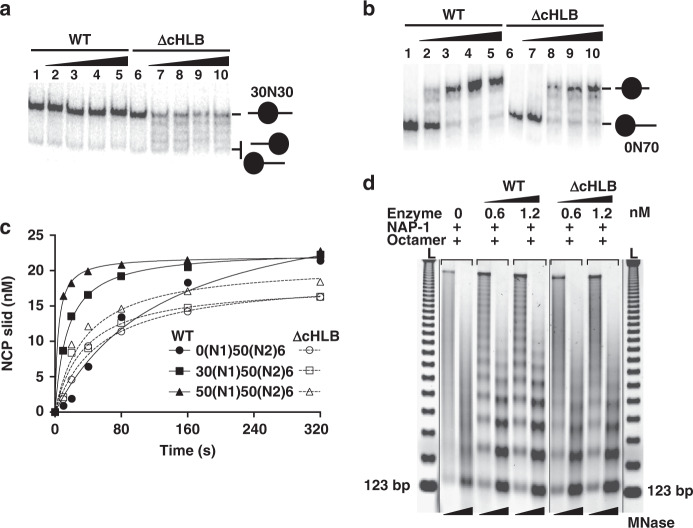
Truncation of the HLB domain relaxes the linker DNA specificity of ISW1a with mononucleosomes and interferes with its nucleosome spacing and dinucleosome remodeling activities. **a**, **b** Gel shift image showing remodeling of (**a**) 30N30 and (**b**) 0N70 mononucleosomes by ISW1a and ISW1aΔcHLB with reaction conditions similar to Fig. 2c. Remodeling assays were from three independent experiments. **c** Plot comparing the amount of dinucleosome remodeled by ISW1a and ISW1aΔcHLB with varying flanking DNA (0-, 30-, and 50-(N1)50(N2)6) over time similar to that shown in Fig. 2e. The estimated rate of dinucleosomes remodeled by ISW1a with 0, 30 and 50 bp of flanking DNA were 0.23, 1.2, and 2.8 nM s^−1^ respectively and the rate of dinucleosomes remodeled by ISW1aΔcHLB with 0, 30, and 50 bp of flanking DNA were 0.33, 0.46, and 0.53 nM s^−1^ respectively. **d** The image of an agarose gel stained with Sypro Gold comparing the spacing activity of wild-type and ΔcHLB ISW1a in the presence of NAP-1 (Nucleosome assembly protein – 1). Source data are provided as a Source Data file.

Linker-DNA dependent remodeling and mononucleosome movement to the center of DNA was blocked by truncation of the HLB domain^22^. Only ∆cHLB ISW1a bound to the SHL-2 position of 43N33 mononucleosomes and mobilized 30N30 nucleosomes (Figs. 5f, g and 6a). Rather than positioning nucleosomes to the center of DNA with 0N70 mononucleosomes like ISW1a, ΔcHLB ISW1a lacked linker DNA sensing and equally distributed nucleosomes along DNA, similar to that observed with 30N30 monucleosomes (Fig. 6a, b, compare lanes 1–5 to lanes 6–10). Truncation of the HLB domain reduced dinucleosome remodeling with 30 and 50 bp flanking DNA, respectively 2.6 and 5.3 times compared to wild-type ISW1a; whereas without flanking DNA there was only marginal differences (Fig. 6c and Supplementary Fig. 7a, b). The residual remodeling activity of ΔcHLB ISW1a is likely due to isolated remodeling of N1 nucleosomes.

Nucleosome spacing by assembling nucleosomes onto linear plasmid DNA with the histone chaperone Nap1, wild-type ISW1a, histones and ATP followed by digestion with micrococcal nuclease was only observed with wild-type ISW1a and not ΔcHLB ISW1a (Fig. 6d^26^). We find that although ΔcHLB ISW1a retained the ability to mobilize mononucleosome, its apparent lack of recognition of dinucleosomes and regulation by DNA linker length abrogates its nucleosome spacing activity.

### High-resolution mapping of nucleosomal DNA movement revealed the specificity of ISW1a for remodeling 50(N1)50(N2)6 dinucleosome and the HLB domain dependency

**Fig. 7:**
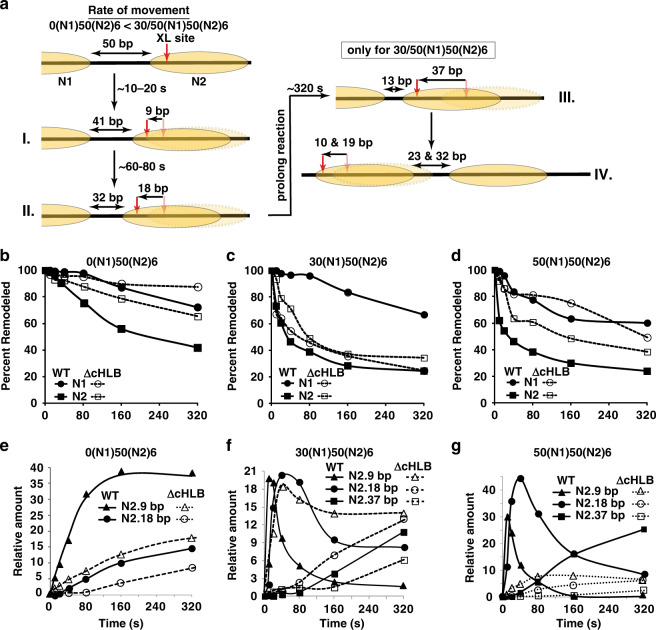
In dinucleosomes, ISW1a is prone to move nucleosome N2 towards N1 and is blocked by truncation of the HLB domain. **a** Schematics show the time course for high-resolution tracking of DNA movement during ISW1a remodeling and spacing of dinucleosomes. Nucleosome movement was tracked by attaching a photoreactive reporter to residue 54 of histone H2B (red arrow) and following where it crosslinks to DNA as dinucleosomes are remodeled. The N1 and N2 nucleosomes are depicted as light tan ellipses. **b**–**d** The extent to which the initial DNA contact is reduced, as observed by loss of DNA crosslinking, was plotted versus time of remodeling for both the N1 (circles) and N2 (squares) nucleosomes. Remodeling was either with wild-type (closed symbol) or ΔcHLB (open symbol) ISW1a. **e**–**g** There are distinct distances to which nucleosomes are moved by ISW1a as detected by crosslinking that were quantitated and plotted versus time for wild type (closed symbol) or ΔcHLB (open symbol) ISW1a. The 9 (triangle), 18 (circle) and 37 bp (square) movement were plotted for N2 nucleosome movement and the type of dinucleosome used is shown above each plot. These experiments had a minimum of three replicates with similar results as those depicted. Source data are provided as a Source Data file.

DNA movement through nucleosomes was followed by replacing residue 54 of H2B with cysteine and coupling a photoreactive probe to cysteine to crosslink and subsequently cleave the most proximal DNA site(s) at different remodeling times [see Fig. 7a and refs. ^27,28^]. The initial movement of N2 nucleosomes by ISW1a was enhanced by 30 and 50 bp of DNA flanking the N1 nucleosomes (Fig. 7a–d, see closed squares versus closed circles and Supplementary Fig. 8a, b) and unlike DNA movement with 0N70 mononucleosomes there was no DNA backtracking. Truncation of the HLB domain dampened ISW1a’s preference to mobilize N2 over N1 nucleosome in the three different dinucleosome substrates (Fig. 7b–d with 0, 30 and 50 bp flanking DNA and Supplementary Fig. 8c) and confirmed earlier DNA footprinting of ISW1a poised to remodel N2 nucleosomes that is lost with the ΔcHLB complex. The 30(N1)50(N2)6 dinucleosome was unusual in that mutation of HLB appeared to enhance remodeling of the N1 nucleosomes to a level comparable to N2, whereas in the other two situations remodeling of N2 was reduced to the level of N1.

We found two early steps in remodeling with DNA having moved 9 or 18 bp from its original position in nucleosomes N2 with wild-type ISW1a and N2 nucleosomes moving faster and farther with 30 or 50 bp of DNA flanking N1 nucleosomes (Fig. 7a, e–g; see closed triangles and circles, stages I and II, and Supplementary Fig. 8b). Movement of 19 bp by N2 nucleosomes positions the two nucleosomes 32 bp apart similar to that observed in our nucleosome spacing assay (Fig. 6d). Even after longer incubation times the spacing distance remained the same with N2 nucleosomes moving 37 bp and N1 nucleosomes moving 10 and 19 bp away from their initial position (Fig. 7a stage III and IV, 7e–g and Supplementary Fig. 8b, c). Truncation of the HLB domain caused N2 nucleosome movement to be significantly slower and reduced compared to wild-type ISW1a, and was most pronounced with 50(N1)50(N2)6 dinucleosomes. The ΔcHLB complex moved the initial step of 9 bp with 30(N1)50(N2)6 dinucleosomes almost as efficiently as wild-type ISW1a, but the next step of 18 bp was clearly defective (Fig. 7f, compare open to closed triangles and circles and Supplementary Fig. 8c, lanes 9–16). In contrast, truncation of HLB impacted more severly both initial steps when remodeling 50(N1)50(N2)6 dinucleosomes (Fig. 7g, compare open to closed triangles and circles and Supplementary Figure 8c, lanes 17–24). Truncation of HLB therefore not only impacted sensing of linker DNA length between nucleosomes, but also adversely effects the ability of ISW1a to recognize 50 bp of DNA flanking the N1 nucleosome, consistent with it binding only one of the two nucleosomes as shown by DNA footprinting.

### Dinucleosome specificity of ISW1a required along with NuA4/SWR1 to regulate transcription

**Fig. 8:**
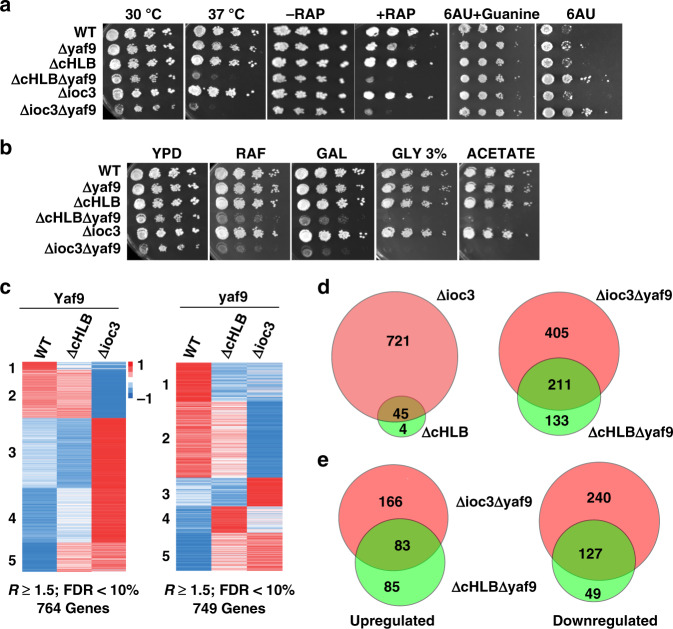
The HLB domain of Ioc3 is required for ISW1a to regulate transcription in conjunction with NuA4/SWR1. **a**, **b** Shown are the changes in cell growth when subjected to **a** heat, Rapamycin (TOR signaling pathway) or 6-azauracil (6 AU) stress or **b** forced to use alternate carbon sources when *IOC3* is mutated or deleted and *YAF9* is deleted. Plates with 6 AU lack uracil and strains contained a centromeric plasmid (PRS416) with URA + marker. The assay had a minimum of three biologically independent samples with results the same as those depicted. **c** The heat map shows gene expression levels for wild-type (WT), *ioc3*∆*cHLB* and *ioc3*∆ in *YAF9* or *yaf9*∆ background strains. Genes with over 1.5-fold change (FC) and false discovery rates (FDR) of <10% compared to wild type were identified and their Z scores plotted for wild-type and mutant ISW1a. The up (red) and down (blue) regulated genes are shown. Data are from three biologically independent samples. **d** A Venn diagram is shown for the overlap of genes differentially regulated in *ioc3*Δ versus *cHLB*Δ and *ioc3*Δ*yaf9*Δ versus *ioc3*Δ*cHLByaf9*Δ with hypergeometric *p* value for the overlap being too small to be determined. In the lower half is shown the Venn diagram of the overlap of upregulated and downregulated genes for *ioc3*Δ*yaf9*Δ *versus ioc3*Δ*cHLByaf9*Δ with hypergeometric *p* value <3.78 *e*−136.

We assessed Ioc3 and HLB function in cells lacking the Yaf9 subunit, because Isw1 genetically interacts with NuA4 and Swr1, whose functions depend on Yaf9^3,29,30^. We found ISW1a genetically interacts with NuA4/SWR1 as the strains *ioc3*∆ *yaf9*∆ and *ioc3*Δ*cHLB yaf9*Δ have a synthetic temperature sensitivity phenotype similar to that previously observed for *isw1*∆ *yaf9*∆ (Fig. 8a)^3^. The *ioc3*Δ*cHLB yaf9*Δ and *ioc3*Δ*yaf9*Δ double mutants were 6-aza-uridine (6 AU) resistant, sensitive to rapamycin and defective in TOR signaling as observed previously for *isw1*Δ*yaf9*Δ (Fig. 8a)^3,4^. We also found that ISW1a is important for using alternative carbon sources other than dextrose using the same double mutants as before (Fig. 8b). Strikingly the defects seen with the *ioc3*Δ*cHLB yaf9*∆ double mutant in all of these assays phenocopied that of the *ioc3*∆ *yaf9*∆ double mutant. These data indicate the in vivo function of ISW1a are dependent on the HLB domain of Ioc3 and the dinucleosome specificity of ISW1a.

RNA-seq revealed both similarities and differences between loss of the ISW1a complex and disruption of ISW1a’s dinucleosome specificity without loss of the complex. Although there was a total of 764 genes misregulated in the *ioc3*Δ strain, there was only a minor transcriptional difference in the *ioc3*Δ*cHLB* strain compared to wild type (Fig. 8c, left side, and 8d, top left). In contrast when either loss of complex integrity or dinucleosome specificity is combined with loss of the Yaf9 subunit of NuA4 and SWR1, both have a significant impact on transcription and are highly similar to each other (Fig. 8c, right side, and 8d, top right and lower part). These data suggest the complete loss of ISW1a alone can impact transcription; whereas loss of the dinucleosome specificity of ISW1a can only significantly impact transcription in conjunction with NuA4/SWR1. Genes downregulated in *ioc3*Δ*cHLB yaf9*∆ and *ioc3*∆*yaf9*Δ strains are involved in the same processes of nucleotide/nucleoside synthesis, metabolism, and respiration and suggest these genes require ISW1A, NuA4 and SWR1 for their activation (Supplementary Table 1). The two heat shock factor encoding genes *HSP12* and *HSP26* were downregulated respectively 35–59 and 14–22 times in the two mutant strains compared to wild type, in agreement with the temperature sensitivity that we have observed. The *IMD2* gene which was previously observed to be upregulated upon the loss of *ISW1* was not significantly changed in *ioc3*Δ*cHLB yaf9*∆ and does not account for it 6 AU resistance^4^. Although there is an overlap of genes that are upregulated in *ioc3*Δ*cHLB yaf9*∆ and *ioc3*∆*yaf9*Δ strains, gene onotology shows a sharp distinction between these two strains. While in *ioc3*Δ*cHLB yaf9*∆ most of the effected genes are involved in transposition, DNA integration and recombination, and other nucleic acid related processes; in *ioc3*∆*yaf9*Δ most of the genes are involved in translation, biosynthesis and metabolism (Supplementary Table 1). We compared our RNA-seq data to the previously published expression profiling of *isw1*Δ *yaf9*Δ using DNA microarrays and found statistically significant similarity, although there are notable transcriptional differences between loss of *ISW1* and that of loss or mutation of *IOC3* (Supplementary Fig. 9). Similar to our current results they observed that metabolism related genes were downregulated in *isw1*Δ *yaf9*Δ; however, genes related to the electron transport chain in their study was upregulated instead of downregulated as we have observed^3^. In contrast to another study, we did not observe ISW1a to repress transcription of the *MET16* gene, but rather we observed that *MET16* expression was 1.7–1.8 times higher in wild type than in *ioc3*Δ*cHLB yaf9*∆ and *ioc3*∆*yaf9*Δ strains^4^.

### The dinucleosome specificity of ISW1a is critical for its proper recruitment and nucleosome spacing activity in vivo

**Fig. 9:**
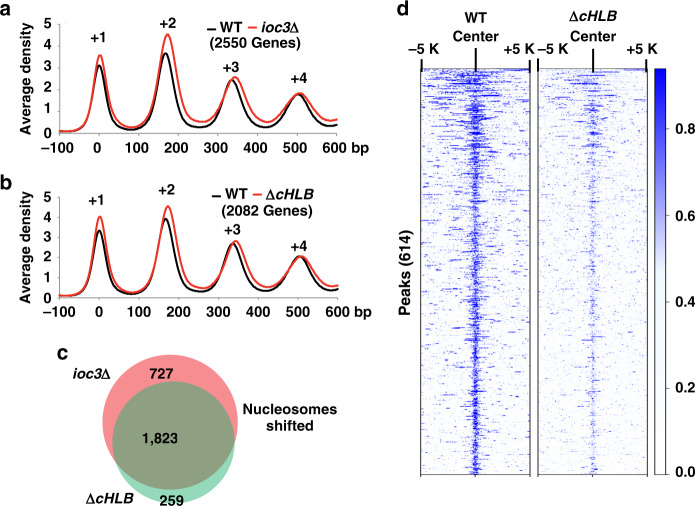
The HLB domain is required globally for proper nucleosome spacing by ISW1a in mRNA coding regions and stable ISW1a binding. **a**, **b** The average nucleosome positioning is shown for those genes in which at least one nucleosome has been significantly shifted (*p* < 0.01) in the +1 to +4 nucleosome coding region for either (**a**) *ioc3*∆ or (**b**) *ioc3∆cHLB* strains (red) compared to WT (black) as detected by MNase-seq. Data are from three biologically independent samples. **c** The Venn diagram shows the extent to which genes with significantly shifted nucleosomes overlap between *ioc3∆* (red) and *ioc3*∆*cHLB* (green). **d** The heatmap is based on 614 high confidence Ioc3 peaks (blue) in wild type and compared wild-type and *ioc3*∆*cHLB* mutant signals at these sites. The data are from biologically independent replicates. The high confidence ChIP-seq peaks of Ioc3 were determined by analyzing with MACS2 (*q* < 0.01).

The direct targets of ISW1a for transcription regulation were identified by determining which of the genes identified by RNA-seq also had ISW1a-dependent positioning of nucleosomes near the transcription start site (TSS) using MNase-seq. MNase-seq was a more sensitive assay for identifying the direct targets of ISW1a than chromatin immunoprecipitation and next-generation sequencing (ChIP-seq) due to the rapid exchange and dynamic nature of ATP-dependent chromatin remodelers on chromatin. We identified >2000 genes in the *ioc3*Δ and Δ*cHLB* strains where nucleosome positioning was shifted downstream of the TSS (*p* < 0.01), particularly at the +2 and +3 nucleosome positions (Fig. 9a, b). The +2 nucleosome was shifted ~10 bp away from the transcriptional start site when either Ioc3 was deleted or the HLB domain was truncated; whereas the +1 nucleosome was not as substantially shifted. The +3 and +4 nucleosomes shifted approximately to the same extent and direction as the +2 nucleosome, suggesting their movement could be in response to changes in the +2 nucleosome position. We found most of the same genes were altered whether nucleosomes were shifted by truncation of the HLB domain or loss of the *IOC3* gene (Fig. 9c). Next, there is significant overlap of those genes that were upregulated or downregulated where nucleosomes are shifted when either the HLB domain is truncated or the Ioc3 subunit is missing (Supplementary Fig. 10a). These data suggest that transcription at a small subset of ISW1a gene targets are either activated or repressed by the coordinated action of ISW1a, NuA4 and SWR1.

The effects after truncating the HLB domain on the stable binding of ISW1a was tested by tagging the C-terminus of *IOC3* and *ioc3*Δ*cHLB* with 13 copies of the Myc epitope and ChIP-seq with anti-Myc antibodies. Truncation of the HLB domain reduced Ioc3 binding at the highly significant Ioc3 peaks identified by ChIP-seq in the wild-type strain, consistent with our earlier biochemical data and without noticeable mis-localization of Ioc3 (Fig. 9d). We found significant peaks at 394 and 131 genes for respectively wild-type and ΔcHLB ISW1a with the majority (106 genes) of them in ΔcHLB ISW1a being the same targets. Further examination of our ChIP-seq data showed that truncation of HLB corresponds to a loss of Ioc3 binding at the +1 and +2 nucleosomes immediately downstream of theTSS (Supplementary Fig. 10), in agreement with earlier Ioc3 mapping studies^1^.

## Discussion

Yeast ISW1a is the first from a large superfamily of ATP-dependent chromatin remodelers to recognize the simplest element of higher-order chromatin organization, namely dinucleosomes. No other member of the ISWI subfamily in yeast or mammals, or the SWI/SNF, CHD or INO80 subfamilies has been found to have dinucleosome specificity even in its closest counterpart, ISW1b, that shares the same catalytic subunit. The only other subunit that ISW1a has besides the catalytic subunit is the Ioc3 accessory subunit and Ioc3 is the key factor contributing to ISW1a’s unique characteristics. The observed preferred dinucleosome configuration for ISW1a of 50 bp DNA flanking the first of the two nucleosomes resembles that seen at promoters with the NFR region and is consistent with earlier Ioc3 ChIP-seq data^1^. By MNase-seq, we also observed that ISW1a moves the +2 nucleosome towards the +1 nucleosome, in full agreement with our biochemical data showing the second nucleosome more distal to the 50 bp of flanking DNA being mobilized by ISW1a. Our observations support prior data showing ISW1a to be sufficient for establishing proper in vivo nucleosome spacing near promoters using chromatin reconstituted in vitro with yeast genomic DNA and remodeled with purified ISW1a^31^.

Allosteric changes in the nucleosomal interactions of ISW1a at the mononucleosome and dinucleosome level appears to be a major factor contributing to the dinucleosome specificity of ISW1a. At the mononucleosome level the allosteric effects are evident when comparing the Isw1 subunit alone versus the ISW1a complex. The active site of Isw1 doesn’t change as it engages at the SHL2 position of nucleosomes and hydrolyzes ATP equally well with Isw1 alone or ISW1a; however the nucleosome mobilizing activity is much less with ISW1a. We show the apparent uncoupling of ATPase activity from nucleosome mobilization being tied to Ioc3 bound at the dyad axis, thereby blocking DNA passage, and Ioc3 also preventing Isw1 from interacting with the acidic pocket of nucleosomes, both of which are required for efficient nucleosome mobilization. We provide further evidence for Ioc3 altering nucleosome mobilization by tracking DNA movement inside nucleosomes and show DNA backtracking proximal to the ATPase domain, likely due to blocking DNA passage at the dyad axis. The backtracking motion is only observed with ISW1a (not Isw1) on mononucleosomes and not on dinucleosomes. The importance of Isw1 contact with the acidic pocket of nucleosomes is consistent with the acidic patch of nucleosomes shown previously to be required for relieving auto-inhibition of mammalian ISWI and a part of the ISWI catalytic subunit outside of the ATPase domain to contact the acidic pocket^16–18^.

There are other allosteric changes observed when comparing ISW1a interactions with dinucleosomes versus mononucleosomes. As before the active site of Isw1 is not changed when ISW1a binds dinucleosomes since it still engages at the SHL2 position of nucleosomes and hydrolyzes ATP comparable to that observed with mononucleosomes, but nonetheless can mobilize dinucleosomes ~10 times faster than mononucleosomes. Differences in remodeling efficiency are accompanied by lifting of the very same interactions that block nucleosome movement when ISW1a is bound to mononucleosomes. When dinucleosome bound, Ioc3 doesn’t bind to the dyad axis of the nucleosome being mobilized by Isw1 and Isw1 is now able to efficiently bind the acidic pocket of nucleosomes. These data raise the question as to what might be the effector in dinucleosomes that causes this allosteric switch. We find by partially truncating the HLB DNA binding domain of Ioc3 that Ioc3 interactions with distal extranucleosomal DNA is essential for efficient ISW1a dinucleosome remodeling. Even though mutant ISW1a retains the ability for the ATPase and HSS domains of Isw1 to properly dock onto mononucleosomes, it loses the ability to recognize dinucleosomes and can only bind to one of the two nucleosomes. The loss of dinucleosome binding cause ISW1a to revert back to less efficiently mobilizing nucleosomes and eliminates nucleosome spacing by ISW1a. We conclude that HLB domain interactions with extranucleosomal DNA serves as an allosteric switch which is critical for nucleosome spacing. Our data suggest an alternative model for nucleosome spacing and linker DNA dependence of ISWI complexes that is not a kinetic dimer switching model as previously proposed^7,8^, but instead involves concerted actions simultaneously between two nucleosomes, the one being moved and the other to which it is being moved towards.

We show that ISW1a works in conjunction with NuA4 and/or SWR1 to regulate transcription both positively and negatively, thus expanding on previous data of Isw1 genetically interacting with these two chromatin remodelers^3^. The co-dependency of these remodelers is connected to stable recruitment of ISW1a to the +1 and +2 nucleosomes and dinucleosome specificity as shown by our Ioc3 mutation experiments. All three complexes work at the +1 and +2 nucleosome positions and are required together to regulate transcription. Questions remains as to the interplay between these three complexes and include how varying nucleosome spacing by ISW1a might influences incorporation of H2A.Z by Swr1 or promote acetylation of H2A.Z by NuA4 which in turn can promote H2A.Z incorporation. H2A.Z is likely to be the linch pin in this coordinated regulation of transcription and has been shown by others to repress or activate transcription and promote RNA polymerase II elongation^32–34^.

Our data support an earlier model proposed for ISW1a that was based on the crystal structures of ISW1a lacking the ATPase domain (Ioc3-HSS) bound to DNA and crosslinking of this truncated ISW1 to two different mononucleosomes^6^. Although this earlier model is consistent with our data, significant parts of the data used to build the model are incongruent with our data. Because the interactions between ISW1a with mononucleosomes and dinucleosomes are so different, it is not accurate to assume the structure of ISW1a bound to dinucleosomes is a summation of the two structures of ISW1a bound to central (43N33) and end (0N70) positioned mononucleosomes as we have observed by DNA footprinting and DNA and histone site-directed crosslinking. Without the ATPase domain in the Ioc3-HSS complex, Ioc3 interactions with nucleosomes is significantly altered compared to wild-type ISW1a with Ioc3 failing to bind nucleosomal DNA at the dyad axis and the lateral face of histone octamer. These interactions and competition with Isw1 are important for the downregulation of nucleosome remodeling when ISW1a is bound to mononucleosomes.

## Methods

### Construction of strains

Two copies of the FLAG epitope was attached to the carboxyl termini of *IOC2* and *IOC3* by PCR to generate tagged ISW1a and ISW1b strains, respectively. The FLAG-tagged *IOC2* and *IOC3* genes were also constructed and cloned into a pET21-cycKanMx plasmid using the NheI and Nde restriction cut sites. The region of *IOC3* encoding amino acids 496 to 565 was deleted using an overlapping PCR based method to construct the pET21-cycKanMx-cHLBΔ plasmid. Plasmids for both *IOC3* and *cHLB*Δ were linearized and transformed into BY4742, transformants selected with kanamycin and verified by PCR amplification of the IOC3 locus and DNA sequencing. *ISW2* was FLAG tagged at its C-terminus^35^.

### Growth assays

Strains were grown to an OD_600_ of 1 in YPD and subjected to a series of 10-fold consecutive dilutions before spotting onto various media. For heat sensitivity test, cells were grown at 30° and 37 °C. To test for defects in the TOR pathway, cells were grown at 24°C on plates containing 100 nM rapamycin. To examine the utilization of various carbon sources, cells were grown at 30 °C on plates containing 2% glucose, 2% raffinose, 2% galactose, 2% potassium acetate and 3% glycerol. To test for possible transcription elongation defects, cells were plated onto -Ura medium containing either 150 µg/ml 6 AU or 150 µg/ml 6 AU + 100 µg/ml guanine and grown at 30 °C. To ensure cells could grow on -Ura medium, they were transformed with a centromeric plasmid containing a URA marker.

### Purification of Wild-type ISWI complexes and mutant ISW1a

Wild-type-ISW2, ISW1b, ISW1a, and mutant cHLBΔ were purified by immunoaffinity chromatography using M2 agarose (Sigma) and eluting with 1 mg/ml FLAG peptide^22,36^. Purity and subunit stoichiometry of each complex were examined by 4–20% SDS–PAGE and staining with Sypro Ruby (Molecular Probes).

### Nucleosome reconstitution, binding, remodeling, and ATPase assays

Mononucleosomes and dinucleosomes were reconstituted with 5.0 µg of PCR-generated DNA or sonicated salmon sperm DNA, 100 fmol ^32^P-labeled probe DNA and 8–10 µg wild-type *Xenopus laevis* histone octamers at 37 °C using a rapid salt dilution method^28^. Oligonucleotides were labeled using Optikinase (USB) and [γ^32^P] ATP (6000Ci/mol^i^) and incorporated into DNA by PCR.

Binding and remodeling were performed at 30 °C in 35 mM Na-HEPES, pH 7.8, 5 mM MgCl_2_, 0.1 mM EGTA, 0.02 M EDTA, 70 mM NaCl, 0.1 mg/ml bovine serum albumin, 5–8% glycerol and 40 μM 2-mercaptoethanol. For binding reactions, enzymes were allowed to bind to the nucleosome substrate for 30 min before analyzing on native 4% polyacrylamide gels in 0.5×TBE and a ratio of acrylamide to bisacrylamide of 35 to 1. Remodeling assays were performed using the same conditions as above and included either ATP or a mixture of ATP and γ-S-ATP as indicated. Reactions were quenched by addition of salmon sperm DNA (2 mg/ml) with either 10 mM γ-S-ATP or 100 mM EDTA and analyzed on native 4% polyacrylamide gels for dinucleosomes, described earlier, or native 5% polyacrylamide gels with an acrylamide to bisacrylamide ratio of 60:1 for mononucleosomes.

The rate of nucleosome-stimulated ATP hydrolysis was measured by preincubating nucleosomes and enzyme for 30 min before addition of 16 μM γ-^32^P-ATP and samples stopped as described after different reaction times. The zero time point had the stop mix add first before addition of ATP. ATP and free phosphate were separated by thin-layer chromatography on polyethyleneimine-cellulose.

### Hydroxyl radical DNA footprinting

ISWI-nucleosome footprints were performed with 2 mM Fe(II)-ammonium sulfate, 0.15% H_2_O_2_, 5.7 mM Na-ascorbate and 2.3 mM EDTA^37,38^. Reactions contained 20 nM nucleosomal substrate and 80 nM enzyme (ISW1a, Isw1, ISW1b, and ISW1aΔcHLB) and the footprinting reaction terminated after 30 s by addition of 100 μl of termination mix containing 5 M ammonium acetate, 5 mM thiourea and 10 mM EDTA. DNA was purified by phenol-chloroform extraction and concentrated by ethanol precipitation at −20 °C before resolving on 6.5% polyacrylamide gel containing 8 M urea. Positions were mapped by comparing to a sequencing ladder generated from the same DNA with Sequenase^TM^ Quick-Denature Plasmid Sequencing Kit from USB. Gels were dried, visualized by phosphorimaging and data analyzed using ImageQuant (V. 5.2) and Microsoft Excel (V. 16.41).

### Site-specific DNA photoaffinity crosslinking

A series of site-specific photoreactive DNA probes for photocrosslinking ISW1a to nucleosomal and extranucleosomal DNA were synthesized using immobilized DNA templates^39^. Biotinylated single-stranded DNA was attached to M-280 Dynabeads and different oligonucleotides hybridized to the template to direct the enzymatic incorporation of photoreactive (AB-dUTP and AB-dCTP) and radioactive ([α-^32^P] dGTP/dATP) nucleotides to a variety of specific sites. After filling in the rest of the DNA with unmodified nucleotides, DNA was released from beads by restriction enzyme digestion. Locations of where the photoreactive and radioactive nucleotides were incorporated into mono- and dinucleosomal DNA are shown in Supplementary Figure S3 and S4. DNA probes for 0N70 and 43N33 were created in a similar way, except that EcoRI was used to excise DNA from the beads for 0N70 and XbaI for 43N33. Due to the expanded length of the dinucleosomal DNA and wanting to scan its breadth with the minimum number of probes, the length of the modified site was expanded to include multiple photoreactive nucleotides per DNA as well as radioactive nucleotides. A total of 11 and 14 different probes were constructed respectively for 0N70 mononucleosomes and 50(N1)50(N2) dinucleosomes. The length of the region modified in the dinucleosomal template ranged from 6 to 30 nucleotides. DNAs were reconstituted into nucleosomes as described earlier and incubated with wild-type or cHLBΔ ISW1a at 30°C for 30 min before irradiation for 3 min at 310 nm, 2.65 mW cm^−2^. The crosslinked nucleosome-enzyme complexes were digested with DNase I and S1 nuclease for transfer of the radioactive label to the crosslinked ISW1a subunits, resolved on 4–20% Tris-Glycine SDS-PAGE and visualized by phosphorimaging.

### Tracking nucleosome movement by histone photocrosslinking to DNA

The DNA site positioned proximal to a unique location on the histone octamer was determined using photoreactive histone octamers^27,36^. Nucleosomes were reconstituted with histone octamer in which residue 53 of histone H2B or 45 of histone H2A was substituted with Cys and later conjugated to *p*-azido phenacyl bromide (APB). DNA movement across the histone octamer surface was tracked during nucleosome remodeling by UV cross-linking at 312 nm for 3 min at different times after ATP was added. The histone-DNA conjugates were purified by phenol-chlorofom extraction and concentrated by ethanol precipitation. DNA was resuspended in 1 M pyrrolidine and the photocrosslinked site cleaved by incubation for 15 min at 90 °C. Pyrrolidine was removed by vacuum drying and cleaved DNA resuspended in loading dye containing 95% formamide. Samples were resolved on 6% polyacrymide gels containing 8 M urea and analyzed as described under the section on hydroxyl radical footprinting.

### Mapping ISW1a interactions with the histone octamer by photocrosslinking

Histone octamers were engineered such that the one naturally occurring cysteine was removed (residue 110 of histone H3) and a single cysteine incorporated at another exposed region of the histone octamer^25^. Seven different histone octamers were made in which residues 19, 89, and 113 of H2A; 44 and 109 of H2B; 80 of H3 or 22 of H4 were replaced with cysteine and conjugated to ^125^I labeled *N*-((2-pyridyldithio)ethyl)-4-azidosalicylamide (PEAS) at room temperature for 30 min. Nucleosome reconstitution and ISW1a binding was as described earlier and sample irradiated at 302 nm for 3 min. The radiolabel was transferred by disulfide reduction with 2-mercaptoethanol, samples separated by SDS-PAGE and stained with Sypro Ruby. The stained gels were dried and phosphorimaged to determine the extent of Ioc3 or Isw1 crosslinking. Signal obtained from each crosslinked subunit was normalized with respect to the percentage of the nucleosome bound to the remodeler and the relative efficiency of ^125^I-PEAS conjugation. The calculated values were plotted using Microsoft Excel (V. 16.41).

### Nucleosome spacing assay

Recombinant *S. cerevisiae* histone octamer (0.5 μg) was mixed with recombinant *S. cerevisiae* Nap1 (1 μg) in the presence of 25 ng/µL BSA, and 0.25% polyvinyl alcohol and polyethylene glycol on ice for 30–60 min to form octamer loading complexes^40^. Relaxed plasmid DNA template was prepared with 3 µg of a 3 kb plasmid and Vaccinia virus Topoisomerase I. Nap1-octamer mix and DNA template were mixed with varying concentrations of wild-type ISW1a or ISW1a cHLBΔ enzymes in the presence of 3 mM ATP and an ATP regeneration system consisting of 3 mM phosphoenol-pyruvate and 20 U/mL Pyruvate Kinase. Reactions were incubated at 30 ^o^C for 2 h. Nucleosome assembly and spacing reactions were digested with 0.6 U micrococcal nuclease (MNase) for 7 min at room temperature and were stopped with 0.1% SDS and 30 mM EDTA. Next, the reactions were digested with 1 mg/ml proteinase K at 65^o^C for 1 hour, extracted with phenol-chloroform and DNA precipitated with ethanol and the addition of 1 M lithium chloride. DNA fragments were resolved using 1.3% agarose gel electrophoresis and stained with ethidium bromide. A 123 bp DNA ladder was used as a molecular weight marker.

### Micrococcal nuclease sequencing (MNase-seq)

Strains with *IOC3-FLAG* and *cHLB*Δ*-FLAG* were grown as three biological replicates at 30 °C in YPD to an OD_600_ of 0.8. Samples were incubated with 1% formaldehyde for 20 min and quenched with glycine (125 mM final) for 5 min^41^. Cells were harvested and converted to spheroplasts using 2 mg/ml zymolase (United Biologicals). Spheroplasts were digested with micrococcal nuclease (NEB M0247S) until ~70% of chromatin was converted into mononucleosomes. Samples were incubated overnight at 65°C with proteinase K (80 μg/μl, Roche 03115879001) and RNase A (30 μg/μl, Thermo Scientific EN 0531), and nucleosomal DNA isolated by phenol:chloroform extraction. DNA was analyzed on a 1.5% agarose gel with mononucleosomal DNA excised and purified using a Qiaquick gel extraction kit (Qiagen 28706). DNA libraries were prepared for Illumina paired-end sequencing of nucleosomal DNA^42^. Samples were paired-end sequenced using a HiSeq 2000 Illumina sequencer and sequence reads were mapped to the sacCer3 reference genome (*Saccharomyces* Genome Database) using Bowtie V. 2.0^43^. Reads that mapped to the repetitive rRNA locus (chrXII: 451275–469084) were filtered out^44^. Dyad density maps were obtained for the mapped reads by considering the center of the paired sequence reads after normalizing to the mean genome-wide coverage. Stereotypic nucleosome positions were identified using a greedy algorithm as described for Gene Track^45^. These nucleosome calls include the nucleosome dyad position, standard deviation of the dyad and nucleosome occupancy values. Nucleosomes with a minimum overlap of 73 bp between wild-type and mutant strains were aligned back to the transcriptional start and end sites (TSS/TES), and were subjected to a Welsh’s *t* test which took into consideration the dyad shift, nucleosome occupancy, and standard deviation of the dyad position of the nucleosome. Nucleosomes were considered to have significantly changed position when *p* < 0.01^1,46^. A total of 5000 genes (*Saccharomyces* Genome Database) larger than 560 bp (containing at least four nucleosomes) and with a well-defined transcriptional start and end sites^47^ were considered for downstream analysis to remove background noise from shorter genes. These genes were aligned based on the position of their +1 nucleosomes and average metagenomic plots comparing wild-type to mutants were generated. The median shift values at each nucleosome position were calculated as described^1^. The data were processed using the web-based genomic data analysis software “Galaxy” (V. 19.01)^48–50^. Further analysis of data was performed in R (V. 3.3.2) and Microsoft Excel (V. 16.41).

### RNA-seq

Yeast strains were grown to an OD_600_ of ~0.7–0.9 in three independent biological replicates. Total RNA was extracted using the hot acid phenol method^51^. Samples were extracted with phenol/chloroform, precipitated with ethanol and resuspended in 50 µl of diethylpyrocarbonate-treated water. After checking the quality of RNA using an Agilent bioanalyzer 2100 and library preparation using the Truseq stranded mRNA library kit (Illumina 20020594, MRZY1324), clusters were generated using cBot followed by 76 bp paired-end sequencing with an Illumina HiSeq 2500. Fragments were mapped to the yeast reference genome sacCer3 using TopHat V. 2.0.10^52^, Bowtie V. 2.0 and R V. 3.1.0^53^. Differential gene expression analysis was performed using edgeR V. 3.6.2^54,55^, limma V. 3.20.4^56^ and htseq-count V. 0.6.0^57^ and the samples were normalized by FPKM. A total of 5,843 annotated genes from the *Saccharomyces* Genome Database were used for analysis. Wild-type and mutant samples were then compared using ≥1.5 fold enrichment and a false discovery rate (FDR) of <10%. The data were clustered (using k-Means, number of clusters = 5) and the mean normalized using Cluster V. 3.0. Heat maps were generated using Java TreeView V. 1.1.6r4^58^. GO term analysis was performed using GOrilla^59,60^. The hypergeometric probabilities for overlapping genes displayed in Venn diagrams (BioVenn V. 1.0.2)^61^ were calculated with the online tool accessed at http://nemates.org/MA/progs/overlap_stats.html and uses the formula $C\left( {D,x} \right) \ast \frac{{c\left( {N - D,n - x} \right)}}{{c\left( {N,n} \right)}}$ where *n* = number of genes in group 1; *D* = number of genes in group 2; *x* = Number of genes in common; *N* = total number of genes and C(value1,value2) = the number of combinations of value1 taken value2 at a time.

### Chromatin immunoprecipitation sequencing (ChIP-seq)

Yeast cultures (100 ml) having a 13xMYC tag at the C-terminus of *IOC3* were grown to an OD_600_ of ~0.8 and formaldehyde crosslinked (1% for 20 min) and quenched with glycine (125 mM final) for 5 min. Cells were resuspended in FA lysis buffer (50 mM Na-Hepes, 140 mM NaCl, 1 mM EDTA, 1% Triton-X, 0.1% Na deoxycholate) containing protease inhibitors (0.5 mM Na Metabisulphite, 1 mM PMSF, 2 μM Pepstatin, 0.6 μM Leupeptin, 2 mM Benzamidine, 2 μg/ml Chymostatin). The cells were lysed using glass beads (zirconia/silica beads, 0.5 mm, BioSpec) after shaking for 1 h at 4 °C. Lysed cells (1 ml) were sonicated using Sonics Vibra Cell 3 mm sonicator [25% capacity, 30 s on 30 s off] for 13 min. After spinning sample at 13,500 rpm, the resulting supernatant was collected. One hundred μl of supernatant was set aside as input sample, while ~450 μl chromatin was incubated overnight at 4 °C with 10 μg anti-myc antibody (Abcam [9E10] ab32) after preclearing for 6 h using magnetic beads. Beads were washed with 1 ml volume of the following solutions: once with chilled FA Lysis buffer, twice with FA-lysis + 140 mM NaCl (50 mM Na-Hepes, 140 mM NaCl, 1 mM EDTA, 0.1% Triton-X), three times with FA-lysis + 500 mM NaC, and twice with LiCl solution (10 mM Tris-HCl pH 8.0, 0.25 M LiCl, 1 mM EDTA, 0.5% NP-40) and eluted with TE + 0.1% NP-40 (10 mM Tris-HCl pH 8.0, 1 mM EDTA, 0.1% NP-40). Samples were treated with RNase A (120 μg/μl) and reverse crosslinked at 65°C overnight with 100 mg/ml proteinase K. DNA fragments were extracted with phenol:chloroform. Libraries were made as in MNase seq and sequenced on a HiSeq 2500. Sequence files were mapped using CASAVA V.1.8.2 (Illumina) and sacCer3 was used as the reference genome. Peaks were called using MACS V.2^62^ with a cutoff *q* < 0.01. The *q* value is an adjusted *p* value and the peak centers were plotted using deepTools V. 2^63^.

### Statistics and reproducibility

The affinity or dissociation constant (*K*_D_) of ISW1a, ISW1b, ISW2 and ΔcHLB ISW1a for mononucleosomes and/or dinucleosomes was determined using EMSA. The estimated K_D_ for each of these was determined using a minimum of *n* = 3 independent experiments and in the graphs the error bars are presented as mean values +/− SD. Confidence intervals are provided for each K_D_ from GraphPad (PRISM V. 6.0b) to show the range of values in which the *K*_D_ has a 95% probability of lying within. The gel images for all binding assays are representative of a minimum of *n* = 3 independent experiments including those shown in Figs. 1f and 2c, d and Supplementary Figs. 1a, b, d, 5d, 6a, and 7a, b. Nucleosome remodeling and ATPase assays were done a minimum of *n* = 3 independent experiments and error bars are presented as mean values +/− SD. The gel images of nucleosome remodeling assays are representative of a minimum of *n* = 3 independent experiments including those shown in Figs. 2c, d and 6a, b and Supplementary Figs. 2a–c, e–i, and 7a, b. Wild-type and ΔcHLB ISW1a were purified a minimum of *n* = 4 independent times and each showed a similar composition as visualized by SDS-PAGE and Coomassie blue staining to that shown in Fig. 5c.

Mapping of histone-DNA interactions using modified histones crosslinked to radiolabeled DNA were repeated in *n* = 3 independent experiments with similar patterns observed over the different time points as shown in Fig. 3b, c and Supplementary Fig. 8a–c. DNA footprinting experiments were repeated in *n* = 3 independent experiments for each condition with different chromatin remodelers and nucleosome substrates, and similar patterns were observed in each replicate like that shown in Figs. 1c–d, 1g, and 5d–g. Nucleosome spacing assays with NAP-1, histone octamer and wild-type or ΔcHLB ISW1a were repeated in *n* = 3 independent experiments and had similar results to that shown in Fig. 6d. DNA and histone photocrosslinking of wild-type and ΔcHLB ISW1a were repeated in *n* = 3 independent experiments and all results were similar to that shown in Supplementary Fig. 5a, b, c, d. The efficiency of DNA and histone crosslinking was quantitated by phosphorimaging relative to an internal standard and plotted with errors bars representing the mean values +/− SD.

### Reporting summary

Further information on research design is available in the Nature Research Reporting Summary linked to this article.

## Supplementary information

Supplementary Info

Peer Review

Reporting Summary

## Data Availability

The data that support this work are available from the corresponding author upon reasonable request. The datsets generated during and analysed during the current study are available in the NCBI’s Gene Expression Omnibus with accession number ‘GSE150829’ The structures of the ISWI-nucleosome complex in an ADP-bound state was obtained from the Protein Data Bank entry ‘6IRO’. Reference genomes (sacCer3, R64-2-1) and gene lists are obtained from the ‘*Saccharomyces* Genome Database. [http://sgd-archive.yeastgenome.org/sequence/S288C_reference/genome_releases/]’. Source data are provided with this paper.

## Source data

Source Data
