# Supplementary material for: Dinucleosome specificity and allosteric switch of the ISW1a ATP-dependent chromatin remodeler in transcription regulation
Source: Nat Commun. 2020 Nov 20;11:5913. doi: 10.1038/s41467-020-19700-1 (PMC7680125; doi:10.1038/s41467-020-19700-1)
Supplement: Supplementary file 1 — Supplementary Info [file 41467_2020_19700_MOESM1_ESM.pdf]

Supplemental Table and Figures

Table 1. Highest significance GO terms for upregulated and downregulated genes in *Ioc3*Δ *yaf9*Δ and *Ioc3*Δ*cHLB yaf9*Δ. The ES symbol refers to enrichment score and the *p* values represent the enrichment *p*-value derived according to mHG or HG model and is not corrected for multiple testing. The FDR values provided in the Source Data represent the corrected *p*-value.

Category	GO name	ES	# Genes	P value
	*ioc3*Δ *yaf9*Δ			
	Upregulated			
Translation	Mitochondrial translation	7.63	34	6.04E-22
	Translation	2.79	38	4.01E-09
	tRNA aminoacylation for mitochondria	8.34	5	2.01E-04
Biosynthesis	Amide biosynthetic process	2.65	43	2.62E-09
	Peptide biosynthetic process	2.76	38	5.23E-09
	Cellular macromolecule biosynthetic process	1.87	55	1.83E-06
	Macromolecular biosynthetic process	1.79	59	2.60E-06
	Organonitrogen compound biosynthetic process	1.78	56	6.05E-06
Metabolism	Cellular amide metabolic process	2.48	46	3.81E-09
	Peptide metabolic process	2.59	39	2.12E-08
	Mitochondrial RNA metabolic process	5.7	10	5.95E-06
Catabolism	Sucrose catabolic process	8.49	4	8.52E-04
Nucleic Acid	Synapsis	10.38	4	3.48E-04
	Carbon catabolite activation of transcription*	5.39	6	6.47E-04
	Carbon catabolite regulation of transcription*	4.3	7	9.76E-04
	Downregulated			
Metabolism & Respiration	Oxidation-reduction process*	3.69	95	2.78E-32
	Drug metabolic process*	3.7	59	7.34E-20
	Small molecule metabolic process*	2.35	108	2.11E-19
	Respiratory electron transport chain*	9.28	19	2.07E-15
	Generation of precursor metabolites & energy*	3.99	39	1.81E-14
	Energy derivation by oxidation of organic compounds*	4.84	30	8.78E-14
	Energy coupled proton transport, down electrochemical gradient*	12	13	4.56E-13
	ATP synthesis coupled proton transport*	12	13	4.56E-13
	Electron transport chain*	6.25	22	5.25E-13
	ATP metabolic process*	6.57	21	5.70E-13
	Cellular respiration*	6.14	22	8.14E-13
	Proton Transmembrane transport*	5.21	25	1.68E-12
	Inorganic ion transmembrane transport *	3.74	33	1.43E-11

	Inorganic cation transmembrane transport*	3.88	31	2.24E-11
	Aerobic respiration*	6.06	19	4.09E-11
	Carbohydrate derivative metabolic process	2.44	46	7.23E-09
Nucleoside & Nucleotide	Ribose phosphate metabolic process*	3.82	34	3.46E-12
	Purine ribonucleoside triphosphate metabolic process*	5.97	21	5.29E-12
	Purine ribonucleotide triphosphate	4.28	29	7.35E-12
	Purine nucleoside triphosphate metabolic process*	5.76	21	7.35E-12
	Ribonucleotide metabolic process	3.81	29	1.62E-10
Ioc3ΔcHLB yaf9Δ				
Upregulated				
Nucleic acid	Transposition, RNA-mediated	11.5	12	1.59E-10
	DNA integration	11.5	12	1.59E-10
	Transposition	10.1	12	8.83E-10
	RNA-dependent DNA biosynthetic process	7.81	12	2.21E-08
	DNA biosynthetic process	5.11	12	2.90E-06
	DNA recombination	3.07	17	3.15E-05
	Carbon catabolite activation of transcription*	7.97	6	7.66E-05
	RNA phosphodiester bond hydrolysis, endonucleolytic	3.37	12	2.06E-04
	Carbon catabolite regulation of transcription*	5.45	6	6.89E-04
Metabolism	Cellular carbohydrate metabolic process	3.14	14	1.30E-04
	Cellular response to nutrient	4.83	7	5.26E-04
Transport	Anion transport	2.88	15	1.98E-04
	Ammonium transport	7.84	5	3.93E-04
	Organic anion transport	3.01	13	3.42E-04
Downregulated				
Respiration & Metabolism	Oxidation-reduction process*	3.71	46	9.58E-16
	Energy coupled proton transport, down electrochemical gradient*	17.2	9	3.97E-10
	ATP synthesis coupled proton transport*	17.2	0	3.97E-10
	Drug metabolic process*	3.65	28	1.38E-09
	Cellular respiration*	7.54	13	8.44E-09
	Aerobic respiration*	7.95	12	1.69E-08
	Generation of precursor metabolites and energy*	4.03	19	1.62E-07
	Inorganic ion transmembrane transport*	4.24	18	1.65E-07
	ATP metabolic process*	7.14	11	2.25E-07
	Serine family amino acid metabolic process*	7.92	10	2.89E-07
	Respiratory electron transport chain*	9.13	9	3.07E-07
	Proton transmembrane transport*	5.63	13	3.39E-07
	Electron transport chain*	6.49	11	6.30E-07
	Inorganic cation transmembrane transport*	4.16	16	1.08E-06

	Energy derivation by oxidation of organic compounds*	4.69	14	1.22E06
	Small molecule metabolic process*	1.94	43	8.26E-06
Nucleotide & Nucleoside	Purine ribonucleoside triphosphate metabolic process*	6.49	11	6.30E-07
	Purine nucleoside triphosphate metabolic process*	6.27	11	9.21E-07
	Ribonucleotide triphosphate metabolic process	5.95	11	1.58E-06
	Ribose phosphate metabolic process*	3.27	14	8.25E-05

* The same GO term is found in both *ioc3*Δ*yaf9*Δ and *ioc3*Δ*cHLB yaf9*Δ

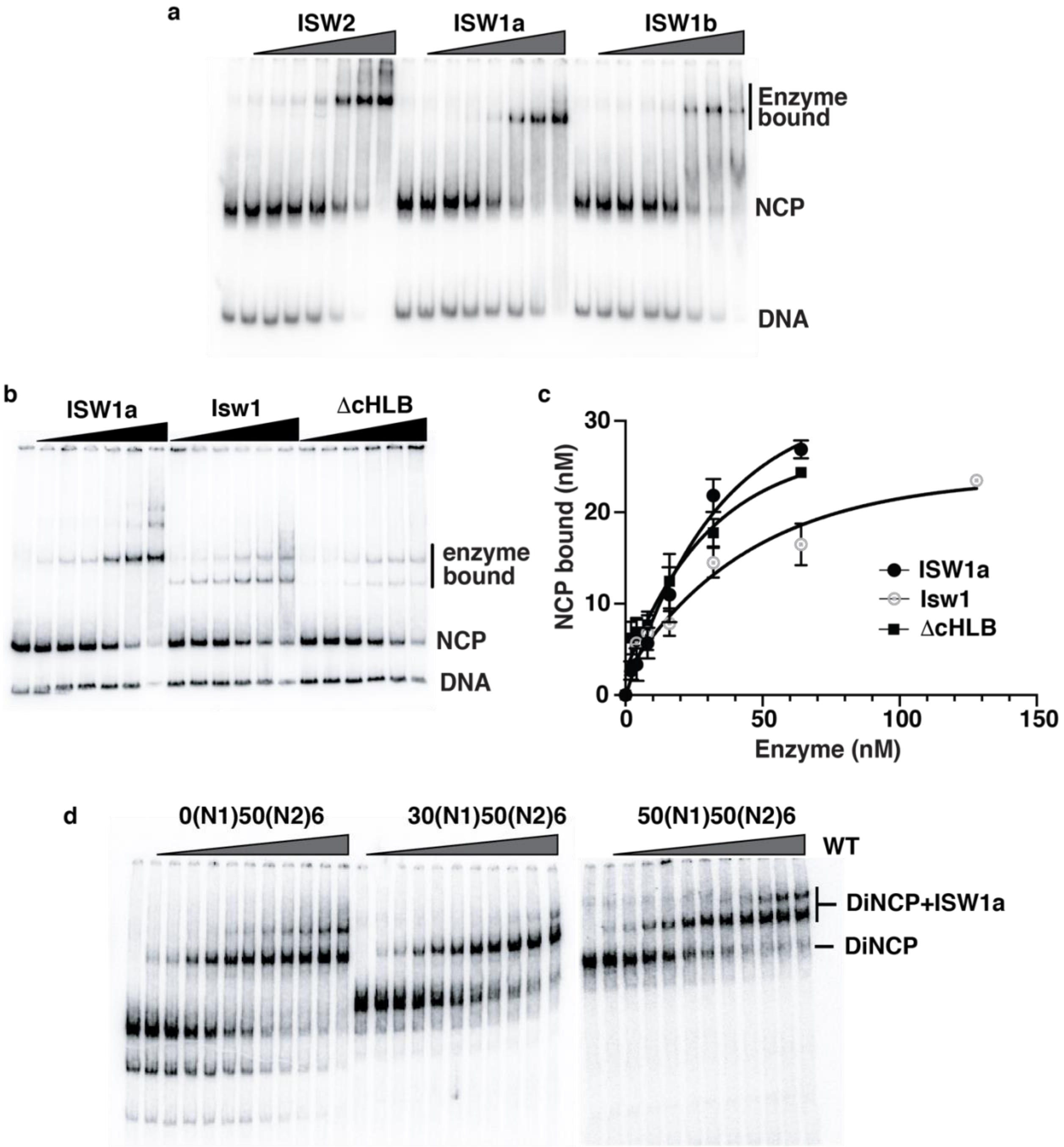

Supplementary Figure 1. ISW1a has a higher affinity for dinucleosomes with 50 bp of flanking DNA and 50 bp of linker DNA.

(a) A representative EMSA is shown for ISW1a, ISW1b and ISW2 binding to 0N70 mononucleosomes and corresponds to that used in Figure 1b. (b) The affinity of ISW1a, Isw1 and ΔcHLB ISW1a for 0N70 nucleosomes was measured by EMSA using 32 nM nucleosomes and enzyme ranging from 4 to 128 nM for Isw1 and from 2 to 64 nM for wild type and ΔcHLB ISW1a. (c) The amount of enzyme bound to 0N70 nucleosomes was plotted from n=3 independent experiments of the type shown in (b) with the error bars depicted as mean values +/- SD. (d) The affinities of ISW1a for 0(N1)50(N2)6, 30(N1)50(N2)6 and 50(N1)50(N2)6

dinucleosomes were measured by EMSA and were used in Figure 1e. Source data are provided as a Source Data file.

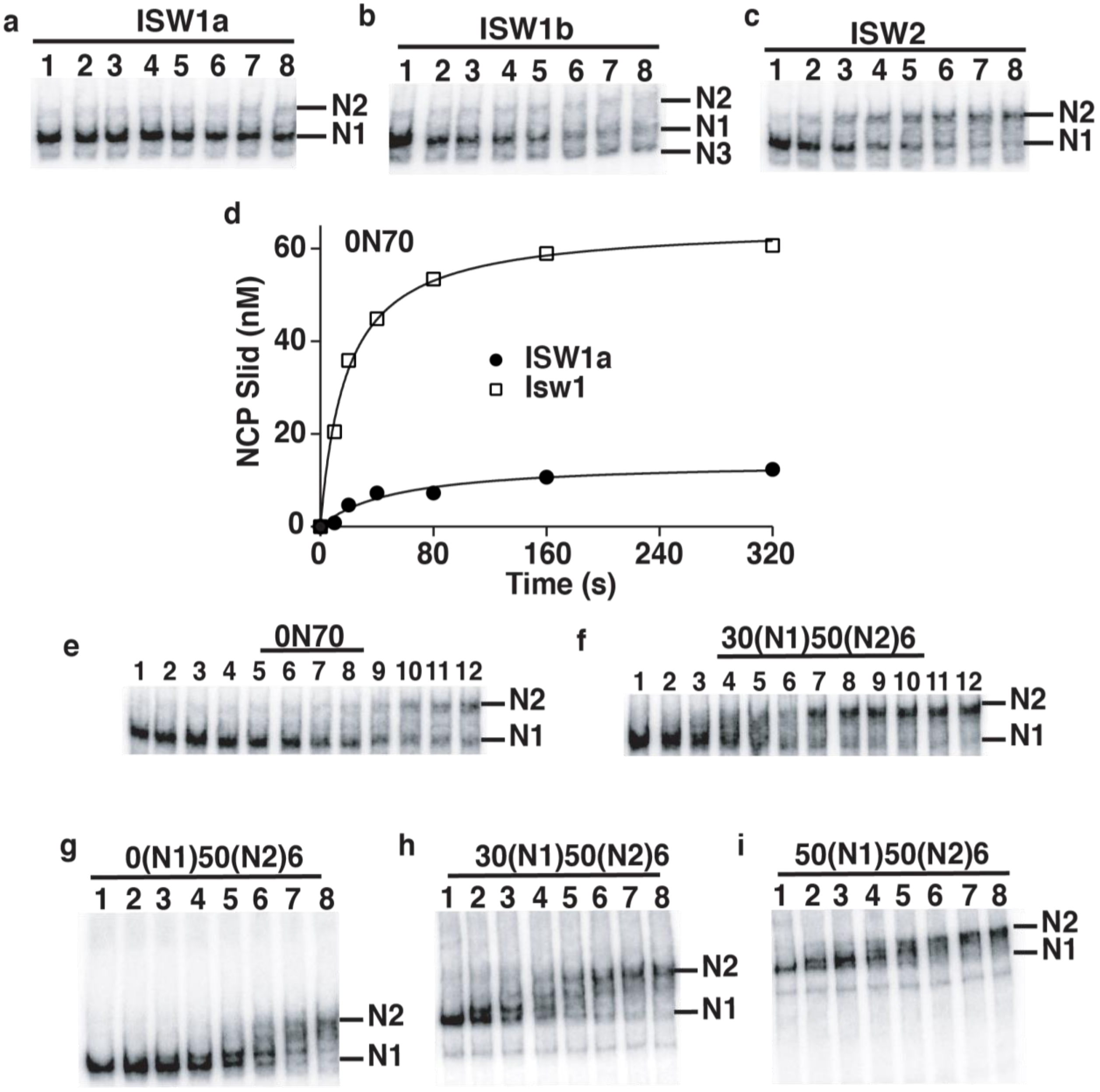

Supplementary Figure 2. The rate of ISW1a remodeling is faster with dinucleosomes than mononucleosomes and ISW1a remodels mononucleosomes slower than Isw1 or ISW1b

(a-c) Rate of 0N70 mononucleosome remodeling with (a) ISW1a, (b) ISW1b or (c) ISW2 was determined by EMSA as shown with reaction times of 0, 5, 10, 20, 40, 80,160 and 320 seconds (refer to Figure 2A). (d) The amount of 0N70 mononucleosome moved by ISW1a or Isw1 was plotted versus time and the initial rate of remodeling by ISW1a and Isw1 was estimated to be 0.25±0.0761 and 3.0±0.13 nM sec^{-i} respectively. Reactions contained 2 μM ATP and correspond to data shown in Figure 2d. (e,f) Examples of the EMSA data used in Figure 2E for determining rates of ISW1a remodeling with (e) 0N70 mononucleosomes and (f) 30(N1)

50(N2)6) dinucleosomes are shown. The reaction times were 0, 5, 10, 20, 40, 80, 160, 320, 640, 1280 and 2560 s. (g-i) Examples of the EMSA used in Figure 2g to determine the rate of ISW1a remodeling with dinucleosomes of varying flanking DNA lengths (0-, 30-, and 50-(N1)50(N2)6) are shown. The reaction times were 0, 10, 20, 40, 80, 160, 320 and 640 s. All gel images are representative of three independent experiments. Source data are provided as a Source Data file.

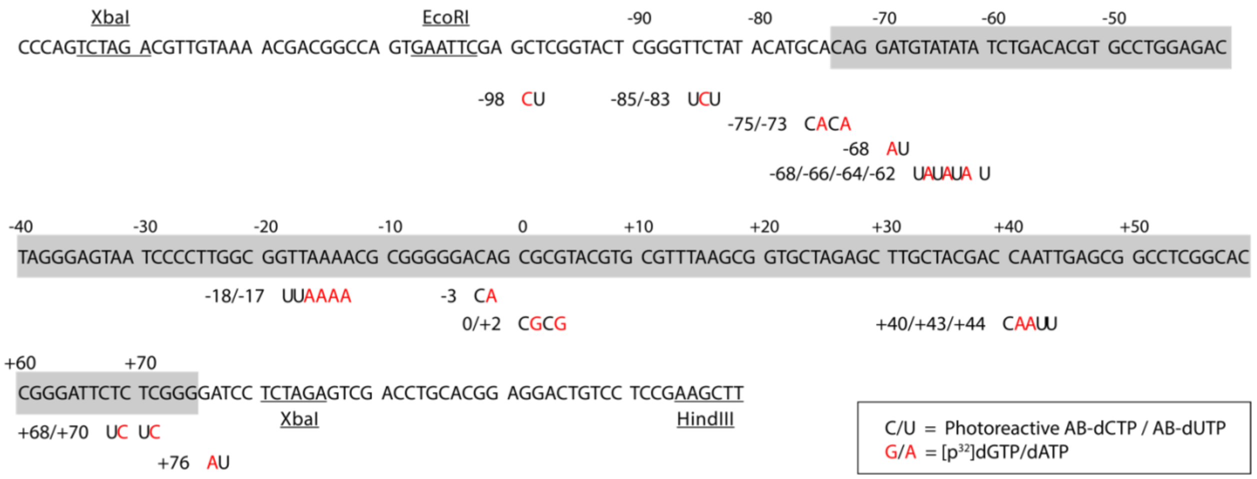

Supplementary Figure 3. Positions of photoreactive and [P^{32}]-radiolabeled nucleotides in mononucleosomes for ISW1a photoaffinity labeling

Shown is the design of the photoreactive DNA probes used in Figure 4a for mapping the DNA interactions of ISW1a bound to mononucleosomes. The reference sequence shows the position of the 601nucleosome positioning sequence in grey. Numbers refer to nucleotide positions relative to the dyad axis (0). Photoreactive nucleotides incorporated were either AB-dCMP or AB-UMP, and radiolabeled nucleotides were either [P^{32}]-dGMP or [P^{32}]-dAMP. Probes for end-positioned (0N70) nucleosomes were released by restriction digestion with XbaI, and for centrally-positioned (33N43) nucleosomes by EcoRI. The HindIII restriction sites marks the end of the DNA template used for probe synthesis.

-90 -80 -70 -60

GAGCTCG GAACGCTATC CGACTGGCAC CGGCACGGTC GCTGTTCGAT ACGTGCGCCG GACGTATATA TCTGACACGT **601b (N1)**

SacI

-95 to -72 (N1) CGGtC GCtGttCGaU aCGtGCGCCG G

-73 to -44 (N1) CCG GaCGUAUAUA UcUGaCaCGt

-50 -40 -30 -20 -10 0 +10 +20 +30 +40

601b (N1) GCCTGGCGAC TAGGGAGCGA TCCCCTTGGC GGTTAAAACG CGGGGGACAG CGCGTACGTG CGTTTAAGCG GTGCTAGAGC TGTCTACGAC CAATTGAGCG **601b (N1)**

GCCtGGCG

-33 to -17 (N1) ggAgCgA UccccUUGGc GGUUaaaa

-10 to +14 (N1) CGGGGGaCaG CGCGUaCGUG cGUUU

+50 +60 +70 -70 -60 -50

601b (N1) GCCTCGGCAC CGGGAATCTC CAGGGCGGCC GCGTATAGGC TCCGGATCCT CTAGAGTCGA CCTCGAAACG GGTACCCCAG GGACTTGAAG TAATAAGGAC **603 (N2)**

+57 to +68 (N1) CaC CGGGaaUcUc cA

+83 to +85(N1) gUAUAgg

-87 to -75 (N2) A CCtCgAAACg ggUA

-73 to -53 (N2) CCCaG GGaCUUgAAg UAAUAAggA

-40 -30 -20 -10 0 +10 +20 +30 +40 +50

603 (N2) GGAGGGCCTC TTTCAACATC GATGCACGGT GGTTAGCCTT GGATTGCGCT CTACCGAGCG CTAAGCGTAC TTAGAAGCGC GAGCGACGAC TTCACACGGT **603 (N2)**

-48 to -29 (N2) aC GGaGGGCCUc UUUcAAcAUc

-18 to -10 (N2) GU GGUUaGCC

-4 to +13 (N2) AUUGCGCt CtaCCGaGCG C

+22 to +49 (N2) tAC ttAGaaGCGC GaGCGaCGAC ttCACAC

+42 to +56 (N2) AC ttCACACGGU

+60 +70

603 (N2) AGGTGGGCGC GCGAACTGGG CAGATCC

aGGUGGG

+60 to +73 (N2) CGC GCGaaCUggg C

C/U = Photoreactive AB-dCTP / AB-dUTP
G/A = $[p^{32}]$dGTP/dATP
a/g/c/t = regulat (unmodified) dNTPs

Supplementary Figure 4. Positions of photoreactive and $[P^{32}]$-radiolabeled nucleotides in 50N50N6 dinucleosomes for ISW1a photoaffinity labeling

Shown is the design of photoreactive DNA probes used in Figure 4b to map the DNA interactions of ISW1a bound to 50(N1)50(N2)6 dinucleosomes. The reference sequence in grey shows the positions of 601b (N1) and 603 (N2) nucleosomes. Numbers refer to nucleotide positions relative to the dyad axes (0) of N1 and N2. Photoreactive nucleotides incorporated were either AB-dCMP or AB-UMP, and radiolabeled nucleotides were either $[P^{32}]$-dGMP or $[P^{32}]$-dAMP. Stretches of modified nucleotides were punctuated with unmodified dNTPs (shown in lowercase). Probes were released by restriction digestion with SacI that cleaves 50 bp upstream of the edge of N1.

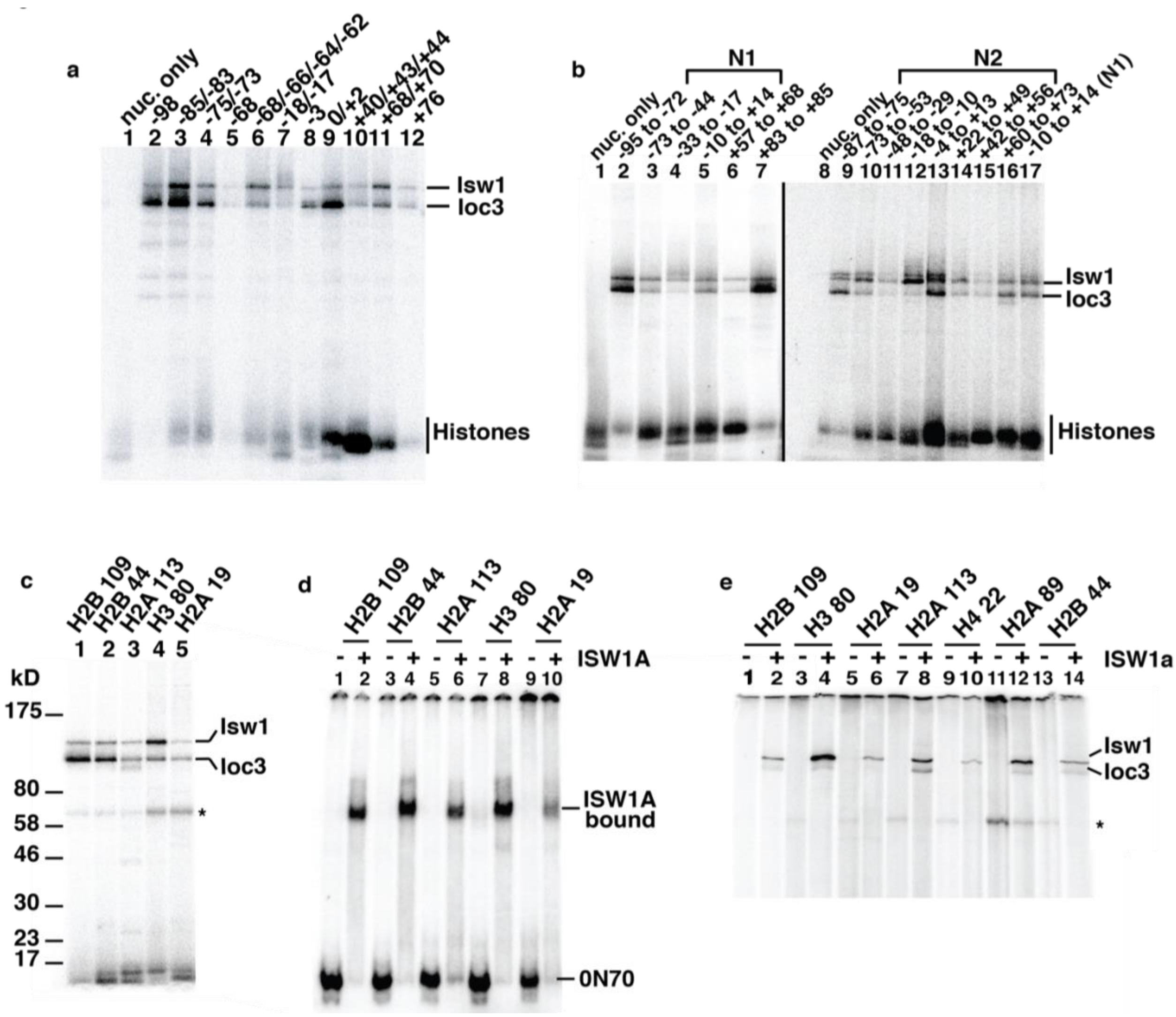

Supplementary Figure 5. Allosteric changes in Isw1 and Ioc3 interactions with dinucleosomes revealed by site-directed DNA and histone crosslinking

(a,b) Representative phosphorimages are shown for DNA crosslinking to ISW1a with (a) 0N70 mononucleosomes and (b) 50(N1)50(N2)6 dinucleosomes. The numbers refer to the position of the photoreactive nucleotides relative to the dyad axis (0). (c and e) ISW1a bound to (c) 0N70 mononucleosomes or (e) 50(N1)50(N2)6 dinucleosomes that were modified at specific histone residues as indicated then photocrosslinked and separated on a 4-20% SDS-PAGE. Crosslinked Isw1 and Ioc3 was detected by phosphorimaging and the * indicates BSA nonspecifically labeled without UV crosslinking. Reactions contained 20 nM of 0N70 mononucleosomes or 50(N1)50(N2)6 dinucleosomes that were ^{125}I labeled at the indicated histone residue. (d) A representative phosphorimage of a native polyacrylamide gel separating free and ISW1a-bound modified 0N70 monucleosomes is shown with ISW1a added in every even lane. Gel images like those shown here were used in Figures 4a-b and 4e-f. All gel images are representative of three independent experiments. Source data are provided as a Source Data file.

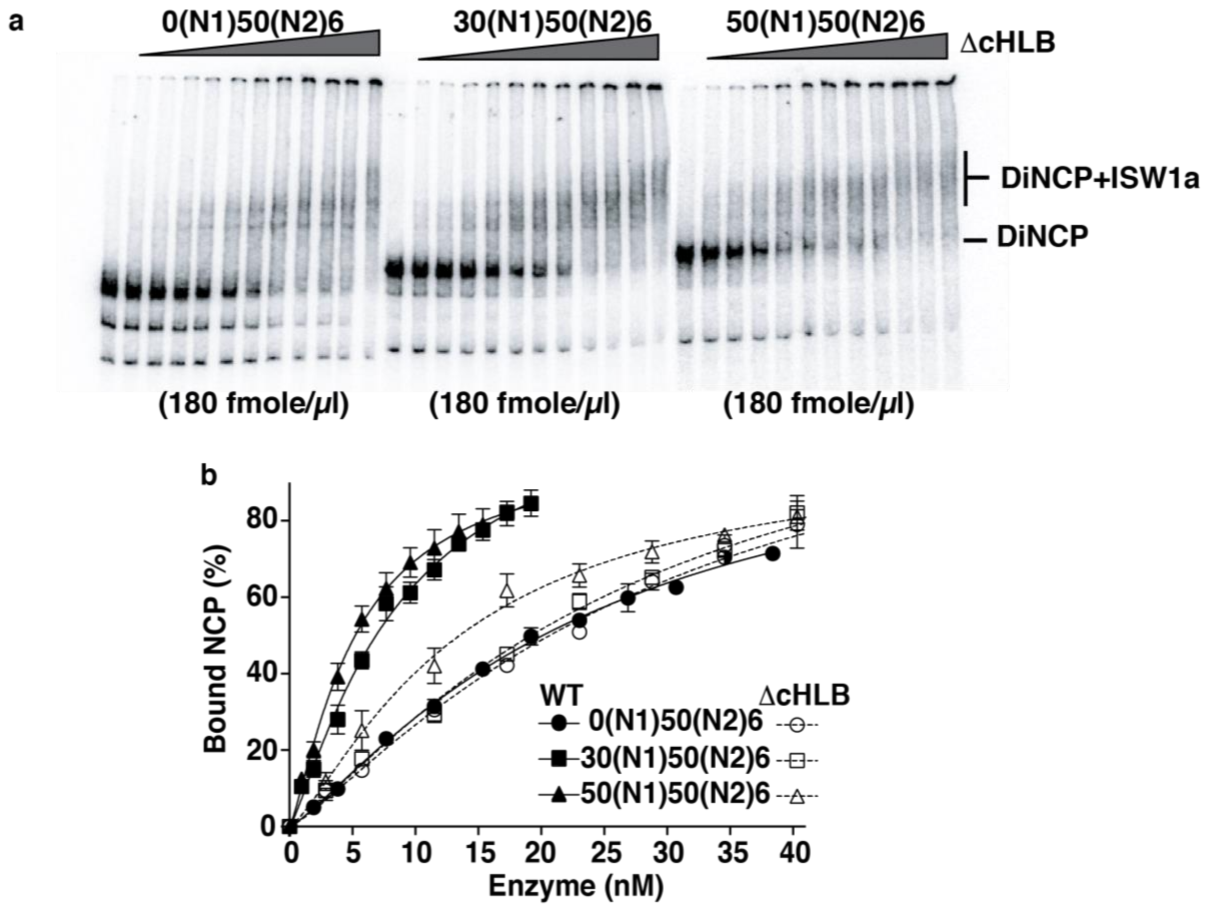

Supplementary Figure 6. The HLB domain of Ioc3 is required for ISW1a's high affinity for dinucleosomes.

(a) The affinities of ΔcHLB ISW1a for 0(N1)50(N2)6, 30(N1)50(N2)6 and 50(N1)50(N2)6 dinucleosomes were measured by EMSA. (b) The extent of 0(N1)50(N2)6 (circle), 30(N1)50(N2)6 (square) and 50(N1)50(N2)6 dinucleosomes (triangle) bound by either wild type (closed symbol) or ΔcHLB (open symbol) were plotted versus enzyme concentration. Data in (b) was analyzed further and the estimated K_D values for wild type and ΔcHLB ISW1a are shown in Table 1.

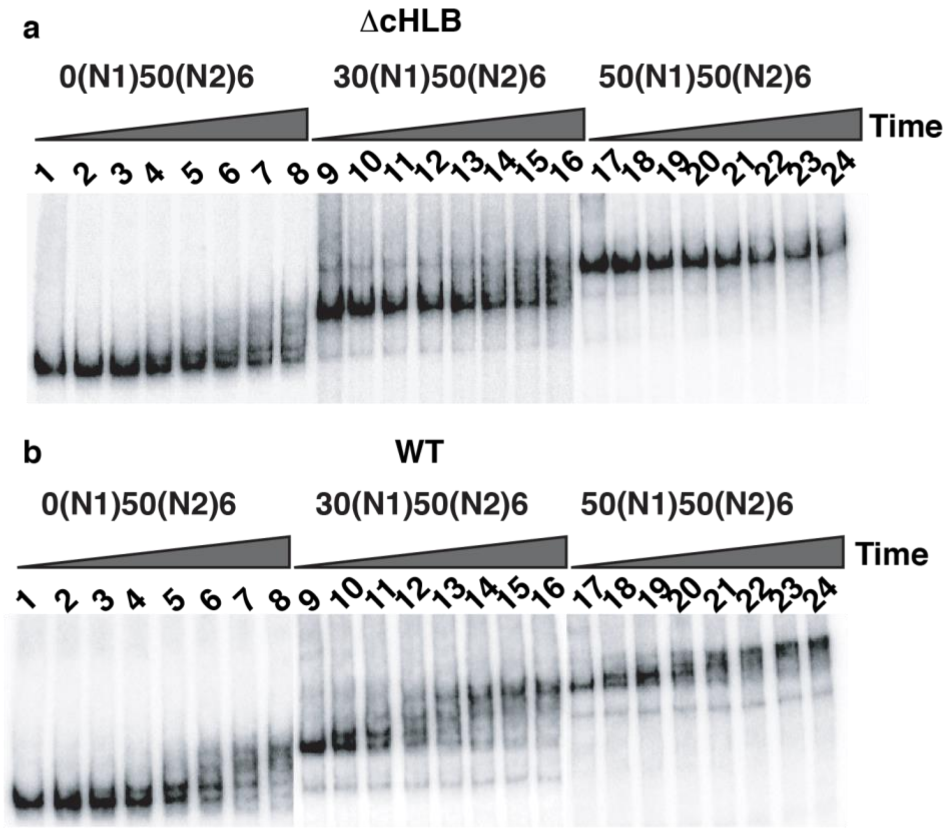

Supplementary Figure 7. Truncation of the HLB domain of Ioc3 interferes with ISW1a ability to mobilize dinucleosomes.

(a,b) Shown are representative gel images of EMSA used in Figure 6c to determine the rate of dinucleosome remodeling with (a) ΔcHLB and (b) wild type ISW1a. Samples had 2 μM ATP and 800 μM γ-S ATP to slow remodeling and reaction times were 0, 10, 20, 40, 80, 160, 320 and 640 seconds. Nucleosomes were fully bound by mutant and wild type ISW1a in these remodeling reactions.

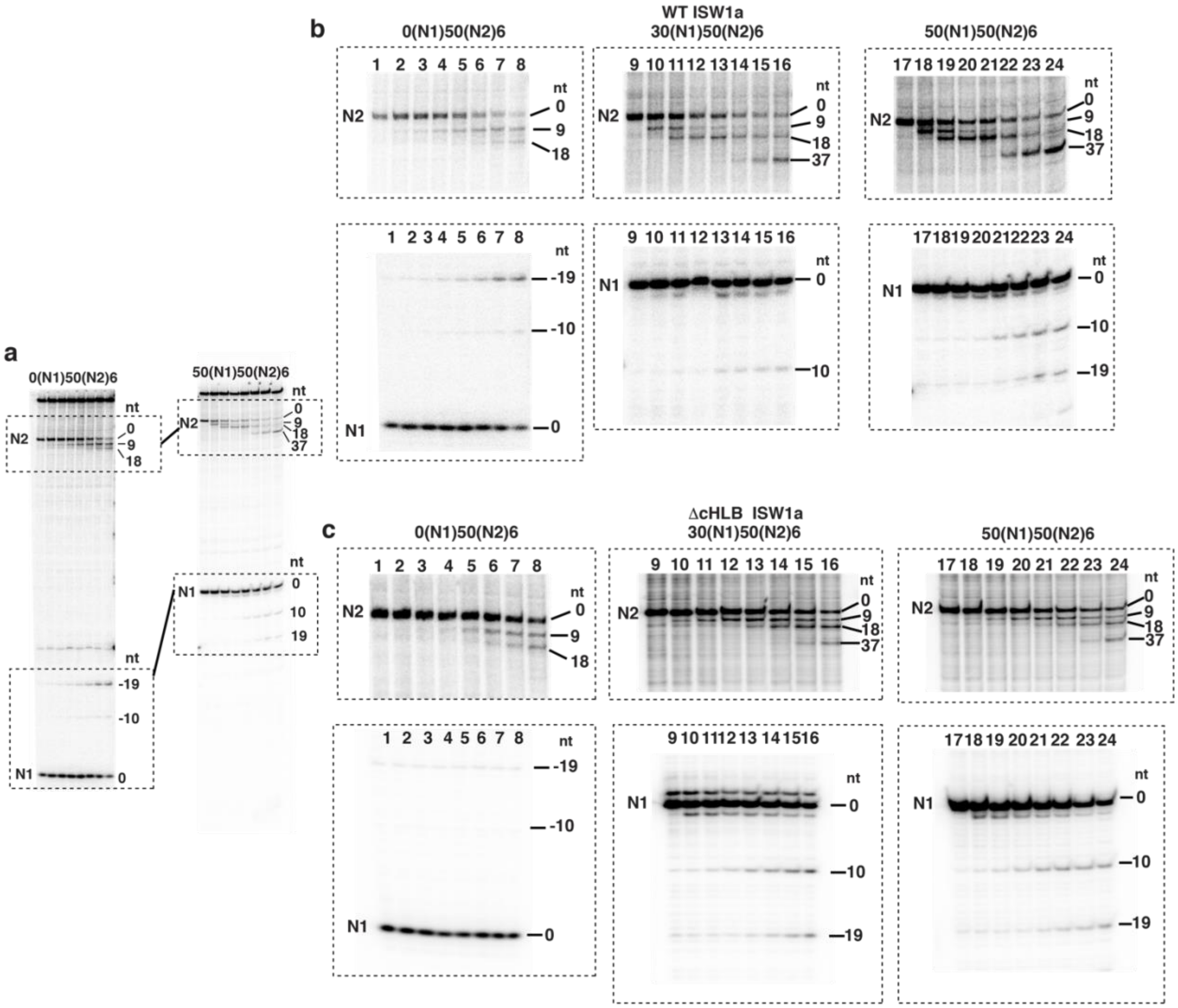

Supplementary Figure 8. Nucleosome movement in dinucleosomes is slower when HLB was truncated.

(a) Example of the gel images used in Figure 7 are displayed for two dinucleosome substrates. The regions that were expanded and analyzed further in (b) and (c) are boxed with dashed lines. The labels on the side indicate which nucleosome is being tracked (N1 or N2) and the number of nucleotides (nt) DNA was moved. (b,c) The expanded regions for the N1 and N2 nucleosomes are shown for three different dinucleosomal substrates as labeled and were remodeled either with (b) wild type or (b) ΔcHLB ISW1a. Source data are provided as a Source Data file.

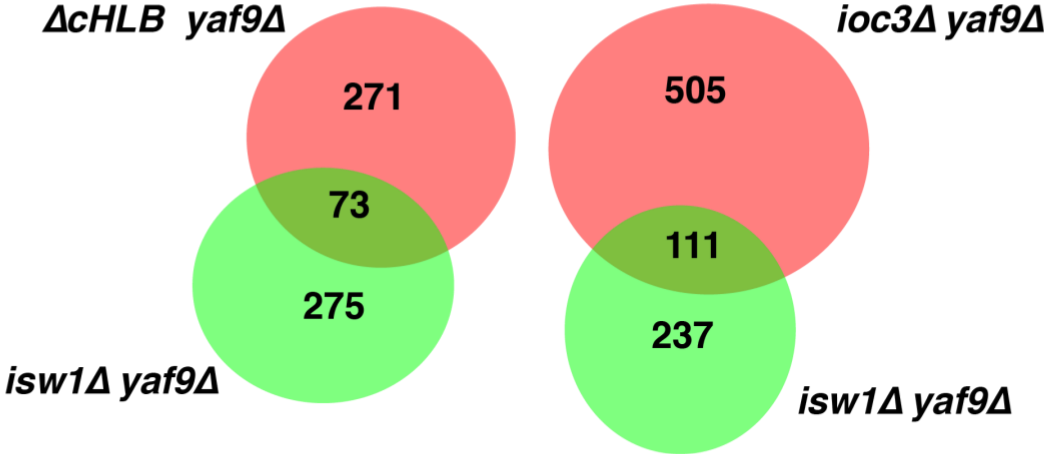

Supplementary Figure 9. There are transcriptional similarities between *isw1*Δ*yaf9*Δ and *ioc3*Δ*yaf9*Δ or *ioc3*ΔcHLB *yaf9*Δ

The Venn diagrams show the extent to which differentially expressed genes detected in our RNA-seq analysis overlap with those from the microarray analysis of Lindstrom et al. 2006. The hypergeometric p value for the left panel is $p < 2.599e\text{-}37$ and for the right panel $p < 2.641e\text{-}57$.

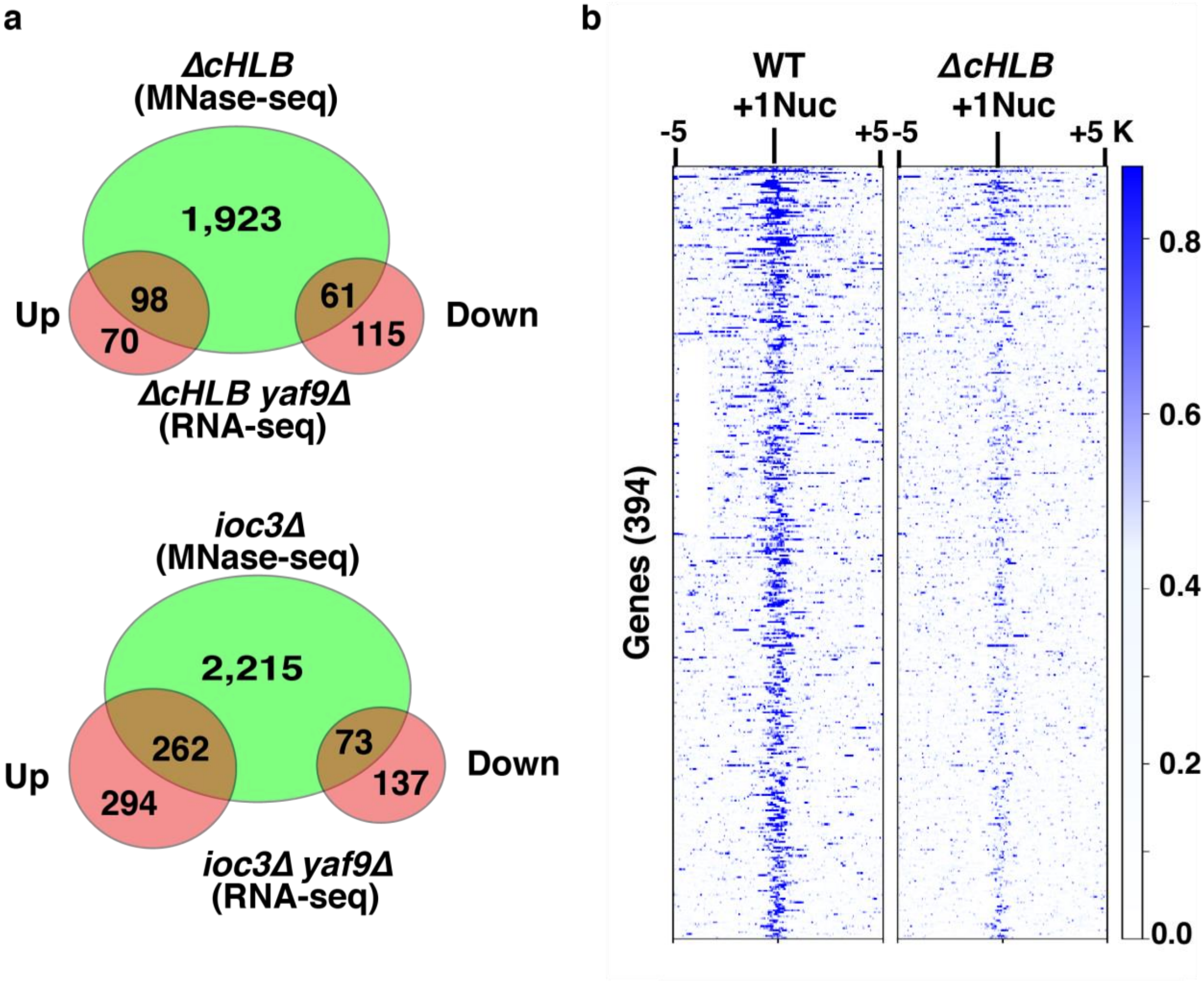

Supplementary Figure 10. Direct transcriptional targets of Ioc3 and the HLB domain were determined using MNase-seq and RNA-seq

(a) Shown is the overlap of the genes whose transcription was upregulated or downregulated to those whose nucleosome positioning was changed by deletion of Ioc3 or truncation of the HLB domain. The hypergeometric *p* values for downregulated *ioc3*Δ*cHLB yaf*9Δ and upregulated *ioc3*Δ *yaf*9Δ were respectively $p<6.76e\text{-}75$ and the others were too small to be measured. (b) Chromatin immunoprecipitation sequencing (ChIP-seq) for Ioc3 found 394 genes where Ioc3 is localized as shown in this heat map centered at the +1 nucleosome positions within a range of ±5 kb region. Binding of Ioc3-ΔcHLB to these genes was greatly diminished as shown in the right-side heat map.
