# Supplementary material for: Dinucleosome specificity and allosteric switch of the ISW1a ATP-dependent chromatin remodeler in transcription regulation
Source: Nat Commun. 2020 Nov 20;11:5913. doi: 10.1038/s41467-020-19700-1 (PMC7680125; doi:10.1038/s41467-020-19700-1)
Supplement: Supplementary file 3 — Reporting Summary [file 41467_2020_19700_MOESM3_ESM.pdf]

nature research

Corresponding author(s): Blaine Bartholomew

Last updated by author(s): Oct 5, 2020

# Reporting Summary

Nature Research wishes to improve the reproducibility of the work that we publish. This form provides structure for consistency and transparency in reporting. For further information on Nature Research policies, see our Editorial Policies and the Editorial Policy Checklist.

## Statistics

For all statistical analyses, confirm that the following items are present in the figure legend, table legend, main text, or Methods section.

| n/a | Confirmed | |
|---|---|---|
| ☐ | ☒ | The exact sample size (*n*) for each experimental group/condition, given as a discrete number and unit of measurement |
| ☐ | ☒ | A statement on whether measurements were taken from distinct samples or whether the same sample was measured repeatedly |
| ☐ | ☒ | The statistical test(s) used AND whether they are one- or two-sided<br>*Only common tests should be described solely by name; describe more complex techniques in the Methods section.* |
| ☒ | ☐ | A description of all covariates tested |
| ☐ | ☒ | A description of any assumptions or corrections, such as tests of normality and adjustment for multiple comparisons |
| ☐ | ☒ | A full description of the statistical parameters including central tendency (e.g. means) or other basic estimates (e.g. regression coefficient) AND variation (e.g. standard deviation) or associated estimates of uncertainty (e.g. confidence intervals) |
| ☐ | ☒ | For null hypothesis testing, the test statistic (e.g. *F*, *t*, *r*) with confidence intervals, effect sizes, degrees of freedom and *P* value noted<br>*Give P values as exact values whenever suitable.* |
| ☒ | ☐ | For Bayesian analysis, information on the choice of priors and Markov chain Monte Carlo settings |
| ☒ | ☐ | For hierarchical and complex designs, identification of the appropriate level for tests and full reporting of outcomes |
| ☒ | ☐ | Estimates of effect sizes (e.g. Cohen's *d*, Pearson's *r*), indicating how they were calculated |

*Our web collection on statistics for biologists contains articles on many of the points above.*

## Software and code

Policy information about availability of computer code

| | |
|---|---|
| Data collection | ImageQuant (V. 5.2) |
| Data analysis | Microsoft Excel (V. 16.41), GraphPad (PRISM V. 6.0b), Bowtie V. 2.0, Galaxy: a web-based genome analysis tool (V. 19.01), R (V. 3.1.0), R (V. 3.3.2), TopHat V. 2.0.10, edgeR V. 3.6.2, limma V. 3.20.4, htseq-count V. 0.6.0, Cluster V. 3.0, Java TreeView V. 1.1.6r4, GOrilla: a tool for discovery and visualization of enriched GO terms (http://cbl-gorilla.cs.technion.ac.il), BioVenn (V. 1.0.2) (https://www.biovenn.nl), http://nemates.org/MA/progs/overlap_stats.html, CASAVA V. 1.8.2, MACS V. 2 and deepTools V. 2 |

For manuscripts utilizing custom algorithms or software that are central to the research but not yet described in published literature, software must be made available to editors and reviewers. We strongly encourage code deposition in a community repository (e.g. GitHub). See the Nature Research guidelines for submitting code & software for further information.

## Data

Policy information about availability of data

All manuscripts must include a data availability statement. This statement should provide the following information, where applicable:

- Accession codes, unique identifiers, or web links for publicly available datasets
- A list of figures that have associated raw data
- A description of any restrictions on data availability

NGS data are available in the NCBI's Gene Expression Omnibus, accession number GSE150829 (https://www.ncbi.nlm.nih.gov/geo/query/acc.cgi?acc=GSE150829). Figures and raw data associated with them are available in the Mendley database at: (https://data.mendeley.com/datasets/pnjsnyh8xb/draft?a=52de0c9e-70da-4e03-b595-d77bff917ff7)
Reference genomes (sacCer3, R64-2-1) and gene lists are obtained from Saccharomyces Genome Database at: (http://sgd-archive.yeastgenome.org/sequence/S288C_reference/genome_releases/)

The structures of the ISWI-nucleosome complex in an ADP-bound state is obtained from Protein Data Bank (PDB-6IRO) at: (https://www.rcsb.org/structure/6IRO)

# Field-specific reporting

Please select the one below that is the best fit for your research. If you are not sure, read the appropriate sections before making your selection.

- [x] Life sciences
- [ ] Behavioural & social sciences
- [ ] Ecological, evolutionary & environmental sciences

For a reference copy of the document with all sections, see nature.com/documents/nr-reporting-summary-flat.pdf

# Life sciences study design

All studies must disclose on these points even when the disclosure is negative.

| | |
|---|---|
| Sample size | For our biochemical assays, independent triplicates were analyzed and the standard deviation from the mean is reported. For biochemical experiments, it is a common practice in the field to use triplicate data to validate the results given the expected variability among samples. Small variability between the triplicates is then ideal to make definitive inferences about biochemical observations and allows distinction between samples. For our in vivo assays, biological triplicates were used to generate independent sequencing data. For ChIP seq, we used biological replicates. Because of sufficient sequence read depth per sample and coverage across each gene is statistically significant, either duplicates or triplicates that are reproducible are accepted in the field. DNA footprinting and mapping histone-DNA contacts experiments had 3 technical replicates and were deemed sufficient when similar patterns were reproducibly obtained. |
| Data exclusions | Data was not excluded. For in vivo datasets (MNAse seq and ChIP seq), once we verified the reproducibility of replicates the files were merged for downstream analysis. |
| Replication | We confirm that our experiments are reproducible and are replicated successfully. |
| Randomization | We have randomly selected biological replicates when applicable. |
| Blinding | All data collection and analyses were blinded from grouping and assumption for unbiased data analyses. |

# Reporting for specific materials, systems and methods

We require information from authors about some types of materials, experimental systems and methods used in many studies. Here, indicate whether each material, system or method listed is relevant to your study. If you are not sure if a list item applies to your research, read the appropriate section before selecting a response.

## Materials & experimental systems

| n/a | Involved in the study | |
|---|---|---|
| [ ] | [x] | Antibodies |
| [x] | [ ] | Eukaryotic cell lines |
| [x] | [ ] | Palaeontology and archaeology |
| [x] | [ ] | Animals and other organisms |
| [x] | [ ] | Human research participants |
| [x] | [ ] | Clinical data |
| [x] | [ ] | Dual use research of concern |

## Methods

| n/a | Involved in the study | |
|---|---|---|
| [ ] | [x] | ChIP-seq |
| [x] | [ ] | Flow cytometry |
| [x] | [ ] | MRI-based neuroimaging |

## Antibodies

| | |
|---|---|
| Antibodies used | anti-myc antibody [9E10] (Abcam ab32) |
| Validation | We have first purified yeast cell lysate (for WT ISW1a and ISW1a lacking cHLB domain - BY4742 strains) and performed western blot analysis (1:1000 dilution). After confirming the 13xMyc tag is present and is detectable using the Myc antibody, we titrated the amount of antibody to use for our ChIP seq experiments. We verified the selectively of the immunoprecipitation using qPCR for positive and negative target regions and optimized our pulldown conditions. After identifying the best conditions, we performed our final ChIP seq experiment. |

## ChIP-seq

### Data deposition

- [x] Confirm that both raw and final processed data have been deposited in a public database such as GEO.
- [x] Confirm that you have deposited or provided access to graph files (e.g. BED files) for the called peaks.

| | |
|---|---|
| Data access links<br>*May remain private before publication.* | (https://www.ncbi.nlm.nih.gov/geo/query/acc.cgi?acc=GSE150829). |
| Files in database submission | MNase seq, RNA seq and ChIP seq data submitted to GEO |
| Genome browser session<br>(e.g. UCSC) | No longer applicable. All data are submitted to GEO database. sacCer3 version of yeast genome was used for mapping. |

## Methodology

| | |
|---|---|
| Replicates | We used two replicates for ChIP seq and once we confirmed peak location and reproducible heat map plots, we merged the two replicated for downstream analysis. MNase and RNA seq data are in triplicates. |
| Sequencing depth | For example, for WT ISW1a input (replicate 2): total number of tags = 6,936,475 and tags mapped to unique location = 5,252927. The read length = 75 bp and reads were single-end. |
| Antibodies | anti-myc antibody [9E10] (Abcam ab32) |
| Peak calling parameters | For peak calling (example WT ISW1a)<br>macs2 callpeak -t ISW1a_IP_replicatecombined_uniq.sorted.bam -c ISW1a_Input_replicatecombined_uniq.sorted.bam -f BAM -g 1.21e7 -n WT_ISW1a_ChIP --outdir /Users/SGHailu/MACS2_Calls -B -q 0.01 --call-summits --extsize 300 --nomodel<br><br>For computing matrix<br>computeMatrix reference-point --referencePoint center -S Merged_WT_ISW1a.bw Merged_dcHLB.bw -R +1Coordinates.bed -a 5000 -b 5000 -o Matrix_ISW1a_wtvsmutant_+1Coordinate_5000bp_sortedby_FC.gz<br><br>For Heat map generation<br>plotHeatmap -m Matrix_ISW1a_wtvsmutant_+1Coordinate_5000bp_sortedby_FC.gz --colorList white,Aliceblue,blue -out Heatmap_ISW1a_ wtvsmutant_+1Coordinate_5000bp_sortedby_FC_Aliceblue.png --zMin 0 |
| Data quality | The resulting peaks were corrected for the pvalue with FDR value of 1%. |
| Software | We have used MACS2 for peak calling and deepTools2 for visualization |
