# Supplementary material for: Dinucleosome specificity and allosteric switch of the ISW1a ATP-dependent chromatin remodeler in transcription regulation
Source: Nat Commun. 2020 Nov 20;11:5913. doi: 10.1038/s41467-020-19700-1 (PMC7680125; doi:10.1038/s41467-020-19700-1)
Supplement: Supplementary file 2 — Peer Review [file 41467_2020_19700_MOESM2_ESM.pdf]

Reviewer #1 (Remarks to the Author):

In this manuscript, Bartholomew and colleagues have compared the substrate specificity of the Isw1a and Isw1b enzymes. They make the remarkable discovery that Isw1a prefers to remodel a dinucleosome substrate rather than a more typical mononucleosome. Importantly, the best dinucleosome substrate also has linker DNA lengths that mimics the organization of the promoter proximal +1 and+2 nucleosomes, the target of Isw1a in vivo. Isw1a has a higher affinity for a dinucleosome, compared to a mononucleosome, which correlates with better remodeling. The authors use a variety of crosslinking approaches to demonstrate that the Ioc3 subunit of Isw1a makes different contacts with a mononucleosome and a dinucleosome. On the mononucleosome, Ioc3 contacts the dyad axis and the octamer lateral surface, supporting a model in which this subunit blocks remodeling of this substrate. The authors identify a DNA binding domain of Ioc3 that contributes to inhibition of mononucleosome remodeling, facilitates remodeling of dinucleosomes, and is important for Ioc3 functions in vivo.

This is a potentially interesting study that helps to explain how Isw1a is targeted to promoter proximal nucleosomes in vivo and how Isw1a activity might be regulated by a novel accessory subunit. Although several foundational pieces of data are very strong, there are technical concerns with some of the experimental setups, and there seems to be a mistake made by the authors regarding how they have interpreted previous in vivo studies, leading to an incorrect rationale for work. This latter issue is easily corrected and does not detract from the significance of the study.

Previous work from the Mellor group (Morillon et al., 2003) defined roles for the yeast Isw1a and Isw1b enzymes in transcriptional control in vivo, using deletion strains for complex-specific subunits. This is the main study cited by the authors. They found that deletion of the IOC3 gene (Isw1a) led to de-repression of the repressed MET16 gene, and their data indicated that Isw1a represses MET16 at the initiation stage, likely by positioning the +1 and +2 nucleosomes. In contrast, they show that loss of Ioc2 or Ioc4 subunits (Isw1b) affects only the active state of MET16 – inactivation of Isw1b leads to a defect in RNAPII phosphorylation, H3-K36me, RNAPII termination, and a change in RNAPII distribution on the activated gene. Unfortunately, the authors have mistakenly stated throughout their paper that Isw1a controls RNAPII transitions during early stages of transcription, a property of Isw1b. The authors need to revise their introduction, results, and discussion to place their data into a context where the role of Isw1a is to position promoter proximal nucleosomes, creating an environment that reinforces transcriptional repression/proper regulation. Note this is also consistent with in vitro data from the Korber group showing that Isw1a is key for +1/+2 positioning. I know of no data supporting a role for Isw1a in transcriptional activation or RNAPII distribution.

In the title and text, the authors argue for an "allosteric switch" for Isw1a. However, I see no evidence for this specific model. Allostery implies that binding of a substrate to one domain/subunit of an enzyme changes how another domain functions. In this case, the Ioc3 subunit clearly makes different contacts on different substrates, but the Isw1 ATPase always engages SHL2 of nucleosome. A more balanced interpretation is that Isw1a has two distinct binding modes. A simple title would be: "Dinucleosome specialization of ISW1a targets remodeling activity to promoter proximal nucleosomes".

Technical issues:

1. The authors show that Isw1a prefers to remodel a dinucleosome substrate, compared to a typical mononucleosome. Remarkably, ATPase activity seems to be identical on both substrates. However, the remodeling and ATPase assays are performed under very different conditions. Remodeling assays have enzyme in excess (20nM Nucs, 80 nM remodeler), whereas ATPase assays typically have nucleosomes in excess. First, the ATPase conditions are not reported here, and it is essential that the methods/legends are updated. Ideally, the remodeling and ATPase assays need to be performed under

similar conditions if the authors want to argue for changes in coupling. This is especially important, given the large changes in affinity between different substrates.

Secondly, in a related point, Figure 2 shows a dramatically weaker affinity of Isw1a for the mononucleosome substrate, compared to the dinucleosome. (1) These approximate Kds need to be reported as done in Fig. S7. Second, it would appear that the mononucleosome affinity is clearly above 50 nM. Thus, it seems unlikely that both di- and mono-nucleosome substrates are fully saturated with enzyme under the current reaction conditions (20 nM substrate, 80 nM nuc). Line 121 in the text states that this is the case, but no data is shown.

2. The EMSA binding data use 20nM nucleosome. However, this is clearly much higher than Kd for the dinucleosome substrate. Consequently, accurate Kd measurements cannot be made, as binding will essentially be stoichiometric. This should at least be mentioned, as the Kds are likely under-estimated.

3. Can the authors eliminate the possibility that the different remodeling activity on various substrates is not due only to changes in affinity? What is the affinity for the catalytic Isw1 subunit for mononucleosomes? This data needs to be added. Is this similar to Isw1a? If so, this would alleviate this concern. This would also speak to the role of Ioc3 – does it weaken binding to mononucleosomes or block activity too?

4. Addition of the 43N33 mononucleosome substrate complicates this story and doesn't seem relevant the dinucleosome specificity. This data can be eliminated. It seems very odd that Ioc3 crosslinks so differently at +3 on this substrate, compared to 0N70, and the dyad contacts are less. This data might be of interest to the remodeler specialist, but it distracts from the main points.

5. It is not clear how the authors have quantified their nucleosome binding assays (e.g. Figure S7). Did the authors use depletion of the free nucleosome or formation of complexes? If the latter, how were the heterogeneous complexes scored? Were gels electrophoresed at rt or 4 degrees? In some cases, loss of Ioc3 seems to lead to smeary, unstable complexes, but the loss of free nucleosome looks similar.

6. The authors model implies that only one copy of Isw1a is bound to either a mononucleosome or dinucleosome. Given evidence that other ISWI complexes (or Chd1) prefer to bind to a single nucleosome as a dimer, is it possible that Isw1a binds cooperatively to dinucleosomes? Binding of two complexes might also explain the hydroxy radical footprinting results, where protection of both "linkers" is observed. Perhaps the authors can titrate Isw1a on a fixed amount of nucleosomal substrate and determine the optimal ratio for remodeling (taking into account binding occupancy)?

7. How were RNA-seq and ChIP-seq signals normalized between samples? Just RPKM? These days it is common practice to include some type of spike-in control for RNA-seq samples to control for library construction. For Ioc3 ChIP-seq, the authors see a general reduction of binding at the promoter nucleosomes. Was this accompanied by increased binding elsewhere or does it reflect poor overall occupancy. The latter case is difficult to interpret without an independent spike-in control.

8. The authors find that a ioc3 yaf9 double mutant shows increased resistance to 6AU. They interpret this result as evidence that Ioc3 (Isw1a) contributes to early elongation control. Typically, 6AU sensitivity (not resistance) is used as a hallmark for a defect in elongation. Furthermore, previous work from the Mellor group (Morillon et al., 2003) showed that isw1 mutants are 6AU resistant because they de-repress the IMD genes, required for purine biosynthesis. This phenotype seems unlikely to be linked to elongation.

9. Line 353-355. The authors state that NuA4 and SWR1 have known roles in early elongation control. There are no references provided, and this reviewer knows of no such data. As shown by the Tsukiyama group (Lindstrom et al., 2006), Isw1 works with NuA4 and SWR1 in transcriptional

repression of stress-induced genes.

Reviewer #2 (Remarks to the Author):

The yeast ATP-dependent chromatin remodeling Isw1 is incorporated into the distinct Isw1a and Isw1b complexes, which localize to different regions of transcribed gene bodies. Isw1a localizes to the 5' regions immediately downstream of the promoter where it might facilitate the transcription from initiation to elongation, but how this might be achieved is unclear. Here, Bhardwaj et al use an array of biochemical and genomic methods to probe the transcriptional functions of the Isw1a complex. Elegant in vitro experiments reveal dinucleosome specificity dependent on its Ioc3 subunit. In vivo genetic and genomic analyses reveal that the HLB domain is the critical portion of Ioc3 function. Taken together, these experiments substantially add to our understanding of the functional distinctions between Isw1a and Isw1b complexes and this study should thus be of significant interest to those in the chromatin and transcription field.

I should qualify these comments by saying that I am not an expert in the biochemistry presented, though the assays seem logical and well controlled, providing clearly interpretable results. Given that my expertise is more on the yeast genomics side of things, my more detailed comments will focus on the data presented in Fig. 8-9. I think the experiments are good, but I think there is more information that can be mined.

-In Fig. 8D, I'm a bit confused by the labeling of the heatmaps. For the left panel, what sample are the WT fold changes calculated against? It seems like there would only be two columns, one for each deletion, with the fold changes calculated against WT for each displayed. I have a similar confusion with the right panel.

-Related to the RNA-seq analysis more generally, is there any correlation between expression level and what genes are dysregulated? Understandably this analysis might be somewhat complicated by indirect effects due to the use of deletions, but I think it's worth a shot.

-Similar to the above, are there any correlations between expression change and nucleosome movement and/or level of Isw1a binding?

-Are the overlaps of genes altered in the current RNA-seq experiments and between the current experiments and previous microarray data (Fig. S10) significant by hypergeometric test or another similar measure? Possibly useful for this: http://nemates.org/MA/progs/overlap_stats.html

-In Fig. 9A, the heatmap is based on 614 Ioc3 peaks. My initial impression is that this was a comparison of WT and mutant signal at 614 Isw1a binding sites detected in WT, but the legend states that there is a "total of 614 differentially localized peaks," which reads to me like only sites showing a significant difference are plotted. Some clarity would be good here.

-Related to the ChIP-seq data, can the authors show some tracks and a heatmap of signal around all TSSs? The heatmap especially would be good to get a sense of the breadth of Ioc3 binding relative to total gene number (and the ChIP-exo data of Yen et al, 2012 Cell).

-I take the numbers shown in Fig. 9B-C (2,550 and 2,082 genes, respectively) to refer to the numbers of genes displaying a significantly shifted nucleosome in the indicated mutant. If so, how does this square with the presumably much smaller number of Ioc3 binding sites detected? Obviously this could be a difference in sensitivity between the two assays, but perhaps worth discussing.

Response to Reviewers' Comments

Reviewer #1 (Remarks to the Author):

In this manuscript, Bartholomew and colleagues have compared the substrate specificity of the Isw1a and Isw1b enzymes. They make the remarkable discovery that Isw1a prefers to remodel a dinucleosome substrate rather than a more typical mononucleosome. Importantly, the best dinucleosome substrate also has linker DNA lengths that mimics the organization of the promoter proximal +1 and+2 nucleosomes, the target of Isw1a in vivo. Isw1a has a higher affinity for a dinucleosome, compared to a mononucleosome, which correlates with better remodeling. The authors use a variety of crosslinking approaches to demonstrate that the Ioc3 subunit of Isw1a makes different contacts with a mononucleosome and a dinucleosome. On the mononucleosome, Ioc3 contacts the dyad axis and the octamer lateral surface, supporting a model in which this subunit blocks remodeling of this substrate. The authors identify a DNA binding domain of Ioc3 that contributes to inhibition of mononucleosome remodeling, facilitates remodeling of dinucleosomes, and is important for Ioc3 functions in vivo.

We thank the reviewer for their appreciation of our delineation of how the ISW1a complex works at the +1 and +2 nucleosomes at promoter regions, the role of the Ioc3 subunit in this process and the intrinsic dinucleosome specificity of ISW1a.

This is a potentially interesting study that helps to explain how Isw1a is targeted to promoter proximal nucleosomes in vivo and how Isw1a activity might be regulated by a novel accessory subunit. Although several foundational pieces of data are very strong, there are technical concerns with some of the experimental setups, and there seems to be a mistake made by the authors regarding how they have interpreted previous in vivo studies, leading to an incorrect rationale for work. This latter issue is easily corrected and does not detract from the significance of the study.

Previous work from the Mellor group (Morillon et al., 2003) defined roles for the yeast Isw1a and Isw1b enzymes in transcriptional control in vivo, using deletion strains for complex-specific subunits. This is the main study cited by the authors. They found that deletion of the IOC3 gene (Isw1a) led to de-repression of the repressed MET16 gene, and their data indicated that Isw1a represses MET16 at the initiation stage, likely by positioning the +1 and +2 nucleosomes. In contrast, they show that loss of Ioc2 or Ioc4 subunits (Isw1b) affects only the active state of MET16 – inactivation of Isw1b leads to a defect in RNAPII phosphorylation, H3-K36me, RNAPII termination, and a change in RNAPII distribution on the activated gene. Unfortunately, the authors have mistakenly stated throughout their paper that Isw1a controls RNAPII transitions during early stages of transcription, a property of Isw1b. The authors need to revise their introduction, results, and discussion to place their data into a context where the role of Isw1a is to position promoter proximal nucleosomes, creating an environment that reinforces transcriptional repression/proper regulation. Note this is also consistent with in vitro data from the Korber group showing that Isw1a is key for +1/+2 positioning. I know of no data supporting a role for Isw1a in transcriptional activation or RNAPII distribution.

We agree with the reviewer and have revised the manuscript accordingly, however there is a problem with some of the data from the Mellor group. We have observed that the *MET16* gene is actually downregulated in our Ioc3 mutants and thus ISW1a appears to have a role in activating this gene. We agree otherwise with the reviewers, but also think there are reasons to consider ISW1a being potentially involved in gene activation. The main reason for this is new to the manuscript. We find that a significant number of genes downregulated in the *ioc3ΔcHLB yaf9Δ* and *ioc3Δ yaf9Δ* to also have Ioc3 and HLB dependent changes in nucleosome positioning, which suggests these are direct rather than indirect targets. Some of these downregulated genes also account for the synthetic temperature and stress sensitivity and

6AU resistance observed in the double mutants..

In the title and text, the authors argue for an "allosteric switch" for Isw1a. However, I see no evidence for this specific model. Allostery implies that binding of a substrate to one domain/subunit of an enzyme changes how another domain functions. In this case, the Ioc3 subunit clearly makes different contacts on different substrates, but the Isw1 ATPase always engages SHL2 of nucleosome. A more balanced interpretation is that Isw1a has two distinct binding modes. A simple title would be: "Dinucleosome specialization of ISW1a targets remodeling activity to promoter proximal nucleosomes".

The definition of allosteric as provide by the reviewer of one domain or subunit changing how another domain function is in full agreement with our data where the Ioc3 alters the way Isw1 functions for remodeling nucleosomes without changing the ATPase activity or engagement of the ATPase domain of Isw1 with nucleosomes. It is also consistent with the definition of allosteric as the regulation of an enzyme by binding an effector molecule at a site other than the enzymes's active site. We assume the reviewer made their conclusions based on the assumption that the remodeling differences we observed were merely due to reduced binding, which is not the case. We have modified the text and figures to more clearly highlight this point. There are three ways in which we observe allosteric effects.

(1). Ioc3 changing the function of Isw1 without effecting the active site of Isw1 at the mononucleosome level.

We show that whether Isw1 alone or in combination with Ioc3 binds to the SHL2 position on mononucleosomes and hydrolyzes ATP equally well, but the ability of the catalytic subunit to mobilize nucleosomes is deeply impacted by the association of Ioc3. We also have additional data in a new Figure 3 showing that ISW1a backtracks or in other words moves DNA in the opposite direction required for moving nucleosomes on to the linker DNA with mononucleosomes, but not with Isw1 alone. DNA movement is thus impacted by Ioc3 even though the ATPase domain is properly engaged on nucleosomes and able to catalyze the hydrolysis of ATP.

(2). An effector site present in dinucleosomes and not in mononucleosomes positively regulates the remodeling efficiency of ISW1a without directly changing the active site of Isw1.

Although ISW1a binds the SHL2 position of nucleosomes of mono- and di-nucleosomes and efficiently hydrolyzes ATP, ISW1a-dinucleosomes are remodeled an order of magnitude more efficiently than mononucleosomes. These data point to an effector site present in dinucleosomes that regulates ISW1a in a manner distinct from that of mononucleosomes. We clearly show the effector site to be distinct from the active site, i.e. ATPase domain, of the Isw1 catalytic subunit.

There are significant conformational changes that occur when ISW1a binds dinucleosomes versus mononucleosomes that don't change the ATPase domain engagement with nucleosomes or hydrolysis of ATP, but instead impacts its mobilization of nucleosomes. We find that Ioc3 only bound the dyad axis in ISW1a-mononucleosome complexes and not with dinucleosomes. We find that Isw1 associates with the acidic pocket of dinucleosomes and doesn't significantly when bound to mononucleosomes. These two conformation changes negatively impact ATP-dependent mobilization of mononucleosomes.

Key differences in DNA movement are also observed with backtracking being observed when ISW1 remodels mononucleosomes and not with dinucleosomes.

(3) We identified a domain of Ioc3 as a part of the effector, which binds distally to linker DNA, doesn't interact with the HAND-SANT-SLIDE domains of Isw1 or alter the binding of Isw1 to the SHL2 of 0N70 nucleosomes. Truncation of this HLB domain alone is sufficient to block docking of ISW1a onto dinucleosomes and points to HLB domain interactions with linker DNA as the key effector for the allosteric changes that occur in ISW1a when bound to dinucleosomes and activating its nucleosome remodeling activity.

It is also true that the interactions of HLB with extranucleosomal DNA impact ISW1a remodeling of mononucleosomes, even though most of ISW1a's interactions with DNA are not changed. The enzymatic activity of ISW1a with 0N70 mononucleosomes is changed when the HLB is truncated so that nucleosomes are not centered on DNA, even though the ATPase domain is bound the same as wild type ISW1a. The enzymatic activity of Isw1 and binding of the ATPase domain with 30N30 mononucleosomes is also changed with truncation of the HLB domain by the ATPase domain engaging the SHL2 position only with the mutant complex and enables remodeling.

Technical issues:

1. The authors show that Isw1a prefers to remodel a dinucleosome substrate, compared to a typical mononucleosome. Remarkably, ATPase activity seems to be identical on both substrates. However, the remodeling and ATPase assays are performed under very different conditions. Remodeling assays have enzyme in excess (20nM Nucs, 80 nM remodeler), whereas ATPase assays typically have nucleosomes in excess. First, the ATPase conditions are not reported here, and it is essential that the methods/legends are updated. Ideally, the remodeling and ATPase assays need to be performed under similar conditions if the authors want to argue for changes in coupling. This is especially important, given the large changes in affinity between different substrates.

We apologize for the confusion about the ATPase assays and the conditions used for this assay. We completely agree with the reviewer that the ATPase and remodeling assays need to be performed under similar conditions and as explained below were indeed performed in this manner. The ATPase assays were generally done under the same conditions as the remodeling assays using excess remodeler versus nucleosomes (see Supplemental Figure S1) and not with nucleosomes in excess as the reviewer thought might be the case. Nucleosome mobilization and ATPase assays contained 20 nM nucleosomes and 80 nM ISW1a with the ATP concentration varying as described in the figure legend. In the comparison of 0N70 mononucleosomes and 30(N1)50(N2)6) dinucleosomes, the ATPase and remodeling reactions both had 16 μM ATP with the key difference being the γ-^{32}P-ATP used in the ATPase reactions. For these reasons, it is possible to conclude there is an uncoupling of ATPase from nucleosome remodeling when ISW1a remodels mononucleosome compared to dinucleosomes. When comparing dinucleosomes with varying flanking DNA lengths, the only difference between the ATPase and remodeling assays is the ATP being added with remodeling assays containing 2 μM ATP and 800 μM γ-S-ATP and the ATPase assays having 40 μM ATP, again as indicated in the figure legend. These details are all provided in the figure legends and the methods section provides the general conditions for the ATPase assay with the comment that the ATP used for each is specified in the respective figure legend. In order to ensure there was no effect due to differences in binding affinity, we confirmed that all ATPase and remodeling assays were done with nucleosomes fully bound by ISW1a using EMSA.

Secondly, in a related point, Figure 2 shows a dramatically weaker affinity of Isw1a for the mononucleosome substrate, compared to the dinucleosome. (1) These approximate Kds need to be reported as done in Fig. S7. Second, it would appear that the mononucleosome affinity is clearly above 50 nM. Thus, it seems unlikely that both di- and mono-nucleosome substrates are fully saturated with enzyme under the current reaction conditions (20 nM substrate, 80 nM nuc). Line 121 in the text states that this is the case, but no data is shown.

We apologize for the confusion caused and have provided additional data clarifying the affinities of ISW1a for the nucleosomal substrates. As the reviewer will see from this data the conditions are indeed fully saturating and the affinity of ISW1a for 0N70 mononucleosomes is actually ~30 nM (Table 1, Figure 1B and Supplemental Figure S1A).

2. The EMSA binding data use 20nM nucleosome. However, this is clearly much higher than Kd for the

dinucleosome substrate. Consequently, accurate Kd measurements cannot be made, as binding will essentially be stoichiometric. This should at least be mentioned, as the Kds are likely under-estimated.

We provide now more analytical data of the EMSA experiments used for determining the K_D values for ISW1a and dinucleosomes by providing the 95% confidence interval and R^2 factor for our data fitting to better evaluate its robustness (Table 1). The concentration of dinucleosomes used in our binding assays is an intermediate one that facilitates in our comparison of the three types of dinucleosomes (dinucleosome concentration = 20 nM and the three K_Ds are 5.29, 10.2 and 45.9).

3. Can the authors eliminate the possibility that the different remodeling activity on various substrates is not due only to changes in affinity? What is the affinity for the catalytic Isw1 subunit for mononucleosomes? This data needs to be added. Is this similar to Isw1a? If so, this would alleviate this concern. This would also speak to the role of Ioc3 – does it weaken binding to mononucleosomes or block activity too?

From the new data added, we show the nucleosome affinity for ISW1a and Isw1 only differ by a slight margin and are fairly comparable (30 vs 22 nM, Supplemental Figure S1B-C and Table 1). When comparing ISW1a vs Isw1 only saturating conditions are used, therefore the observed differences are due to enzymatic and not binding differences.

4. Addition of the 43N33 mononucleosome substrate complicates this story and doesn't seem relevant the dinucleosome specificity. This data can be eliminated. It seems very odd that Ioc3 crosslinks so differently at +3 on this substrate, compared to 0N70, and the dyad contacts are less. This data might be of interest to the remodeler specialist, but it distracts from the main points.

We have eliminated some of the 43N33 mononucleosome data such as histone crosslinking, but have retained the DNA footprinting data as it facilitates in understanding the changes that occur when the HLB domain is truncated both with mono- and dinucleosomes and the allosteric switch. Binding of the ΔcHLB ISW1a complex to 43N33 mononucleosomes resembles that of the same complex binding to the N1 nucleosome in dinucleosomes.

5. It is not clear how the authors have quantified their nucleosome binding assays (e.g. Figure S7). Did the authors use depletion of the free nucleosome or formation of complexes? If the latter, how were the heterogeneous complexes scored? Were gels electrophoresed at rt or 4 degrees? In some cases, loss of Ioc3 seems to lead to smeary, unstable complexes, but the loss of free nucleosome looks similar.

Gel electrophoresis was performed at room temperature and as seen in Figure S7 for wild ISW1a provides discrete bound bands without smearing. We do see smearing with the ΔcHLB complex when bound to nucleosomes and agree that this likely indicates these complexes are less stable to electrophoresis than wild type ISW1a. The amounts of nucleosomes bound were quantified by the disappearance of the free nucleosome band.

6. The authors model implies that only one copy of Isw1a is bound to either a mononucleosome or dinucleosome. Given evidence that other ISWI complexes (or Chd1) prefer to bind to a single nucleosome as a dimer, is it possible that Isw1a binds cooperatively to dinucleosomes? Binding of two complexes might also explain the hydroxy radical footprinting results, where protection of both "linkers" is observed. Perhaps the authors can titrate Isw1a on a fixed amount of nucleosomal substrate and determine the optimal ratio for remodeling (taking into account binding occupancy)?

Unlike recombinant mammalian Snf2h or ACF complexes, we showed a number of years ago that the ISW1a complex exists in solution as complex of only one copy of each subunit and is not a dimer

(Gangaraju et al., Mol. Cell. Biol **8**:3217-25[2007]). We also show now the Hill co-efficient for ISW1a binding to dinucleosomes, which is consistent with a monomer binding to dinucleosomes rather than as a dimer. Furthermore, the footprint pattern of ISW1a bound to dinucleosomes is inconsistent with two monomers of ISW1a, each one binding to one nucleosome, as the footprint for each of the two nucleosomes is very distinct from the other. The N1 nucleosomes has no binding detected at either SHL +/-2 or at SHL +/- 6 and 7, whereas it is observed at these positions in the N2 nucleosome (see Figure 3C).

7. How were RNA-seq and ChIP-seq signals normalized between samples? Just RPKM? These days it is common practice to include some type of spike-in control for RNA-seq samples to control for library construction. For Ioc3 ChIP-seq, the authors see a general reduction of binding at the promoter nucleosomes. Was this accompanied by increased binding elsewhere or does it reflect poor overall occupancy. The latter case is difficult to interpret without an independent spike-in control.

The RNA-seq samples were normalized by FPKM, which is now clearly stated in the methods. When MACS2 analysis for Ioc3 ChIP-seq is performed with wild type and ΔcHLB ISW1a, we found significant a number of peaks for wild type ISW1a located in 399 genes and in 131 for the mutant. The majority of the mutant peaks (106 genes) are the same as those in the wild type sample. These data indicate that Ioc3 binding is not being shifted to other sites in the case of the mutant, but rather only being reduced at the wild type genomic sites.

8. The authors find that a ioc3 yaf9 double mutant shows increased resistance to 6AU. They interpret this result as evidence that Ioc3 (Isw1a) contributes to early elongation control. Typically, 6AU sensitivity (not resistance) is used as a hallmark for a defect in elongation. Furthermore, previous work from the Mellor group (Morillon et al., 2003) showed that isw1 mutants are 6AU resistant because they de-repress the IMD genes, required for purine biosynthesis. This phenotype seems unlikely to be linked to elongation.

We are in complete agreement with the reviewer and have modified the text accordingly. We also find IMD2, the gene observed in Morillon et al 2003, to not be significantly changed in our *ioc3ΔcHLB yaf9Δ* strain compared to wild type (log2 FC of 0.224), contrary to the effects seen by the Mellor group.

9. Line 353-355. The authors state that NuA4 and SWR1 have known roles in early elongation control. There are no references provided, and this reviewer knows of no such data. As shown by the Tsukiyama group (Lindstrom et al., 2006), Isw1 works with NuA4 and SWR1 in transcriptional repression of stress-induced genes.

We are in total agreement and have revised the text accordingly.

Reviewer #2 (Remarks to the Author):

The yeast ATP-dependent chromatin remodeling Isw1 is incorporated into the distinct Isw1a and Isw1b complexes, which localize to different regions of transcribed gene bodies. Isw1a localizes to the 5' regions immediately downstream of the promoter where it might facilitate the transcription from initiation to elongation, but how this might be achieved is unclear. Here, Bhardwaj et al use an array of biochemical and genomic methods to probe the transcriptional functions of the Isw1a complex. Elegant in vitro experiments reveal dinucleosome specificity dependent on its Ioc3 subunit. In vivo genetic and genomic analyses reveal that the HLB domain is the critical portion of Ioc3 function. Taken together, these experiments substantially add to our understanding of the functional distinctions between Isw1a and Isw1b complexes and this study should thus be of significant interest to those in the chromatin and transcription field.

I should qualify these comments by saying that I am not an expert in the biochemistry presented, though the assays seem logical and well controlled, providing clearly interpretable results. Given that my expertise is more on the yeast genomics side of things, my more detailed comments will focus on the data presented in Fig. 8-9. I think the experiments are good, but I think there is more information that can be mined.

1. In Fig. 8D, I'm a bit confused by the labeling of the heatmaps. For the left panel, what sample are the WT fold changes calculated against? It seems like there would only be two columns, one for each deletion, with the fold changes calculated against WT for each displayed. I have a similar confusion with the right panel.

Genes with over 1.5-fold change relative to wild and false discovery rates of less than 10% were identified and their normalized expression values were displayed as Z scores in the heat map. The figure legend has been modified for better clarification.

2. Related to the RNA-seq analysis more generally, is there any correlation between expression level and what genes are dysregulated? Understandably this analysis might be somewhat complicated by indirect effects due to the use of deletions, but I think it's worth a shot.

The requested analysis is shown below. We find a ponderance for the upregulated genes in both the *ioc3ΔcHLB yaf9Δ* and *ioc3Δ yaf9Δ* to be genes that are normally expressed at very low levels, particularly for *ioc3ΔcHLB yaf9Δ*. There is less of a gene expression basis for genes downregulated in *ioc3ΔcHLB yaf9Δ*. We didn't add this data to the main text because it didn't add sufficient information to warrant its inclusion.

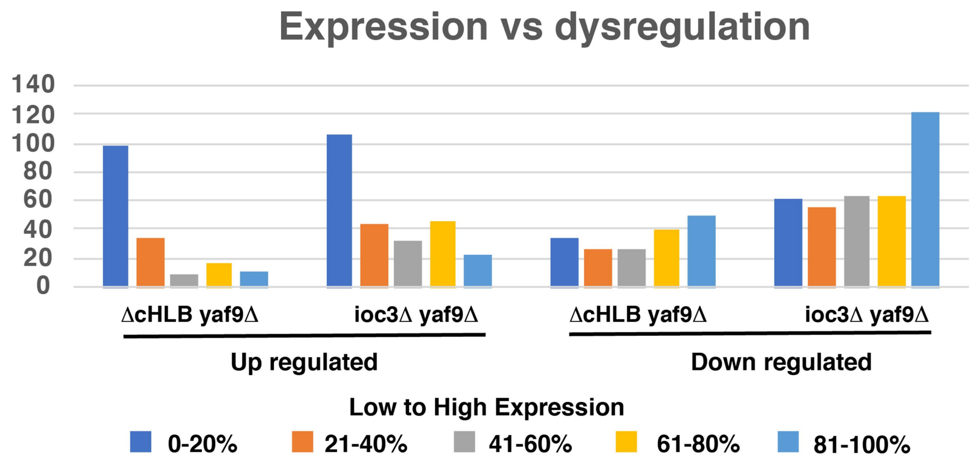

3. Similar to the above, are there any correlations between expression change and nucleosome movement and/or level of Isw1a binding?

We appreciate the reviewer's suggestion and have used Ioc3 and HLB dependent nucleosome positioning to better assess the direct targets in our RNA-seq. As we now show in Supplemental Figure S10A there is a significant overlap of genes that are upregulated and downregulated in *ioc3ΔcHLB yaf9Δ* and *ioc3Δ yaf9Δ* that also have ISW1a dependent changes in nucleosome positioning. The same kind of correlation was not possible with the Ioc3 ChIP-seq data, which was attempted.

4. Are the overlaps of genes altered in the current RNA-seq experiments and between the current

experiments and previous microarray data (Fig. S10) significant by hypergeometric test or another similar measure? Possibly useful for this:http://nemates.org/MA/progs/overlap_stats.html

We now show the significance of Venn diagrams using the hypergeometric test as recommended by the reviewer.

5. In Fig. 9A, the heatmap is based on 614 Ioc3 peaks. My initial impression is that this was a comparison of WT and mutant signal at 614 Isw1a binding sites detected in WT, but the legend states that there is a "total of 614 differentially localized peaks," which reads to me like only sites showing a significant difference are plotted. Some clarity would be good here.

The 614 peaks are based only on wild type and not the difference between wild type and mutant. The high confidence peaks were determined using MACS2 with a threshold q value of <0.01. We show the ChIP-seq signal for wild type and mutant in the vicinity of these 614 peaks. We have modified the figure legend to clarify and avoid the confusion caused previously as noted by the reviewer.

6. Related to the ChIP-seq data, can the authors show some tracks and a heatmap of signal around all TSSs? The heatmap especially would be good to get a sense of the breadth of Ioc3 binding relative to total gene number (and the ChIP-exo data of Yen et al, 2012 Cell).

We do not show the ChIP-seq signal for all genes in heat map format, because generally there is not reliable Ioc3 ChIP-seq signal at most of the genes and is the reason why there is no heat map provided for Ioc3 or Isw1 in the Yen et al, 2012 Cell paper.

7. I take the numbers shown in Fig. 9B-C (2,550 and 2,082 genes, respectively) to refer to the numbers of genes displaying a significantly shifted nucleosome in the indicated mutant. If so, how does this square with the presumably much smaller number of Ioc3 binding sites detected? Obviously this could be a difference in sensitivity between the two assays, but perhaps worth discussing.

We agree with the reviewer that the difference in the numbers of genes detected by MNase-seq and ChIP-seq is likely a reflection of the sensitivity of the two techniques. Chromatin remodelers are generally more difficult to detect by ChIP-seq because their interactions with chromatin are highly transitory and exchange quickly off from chromatin. We have now added a brief discussion of this in the text and the figure below shows the overlap and significance between MNase-seq and Ioc3 ChIP-seq.

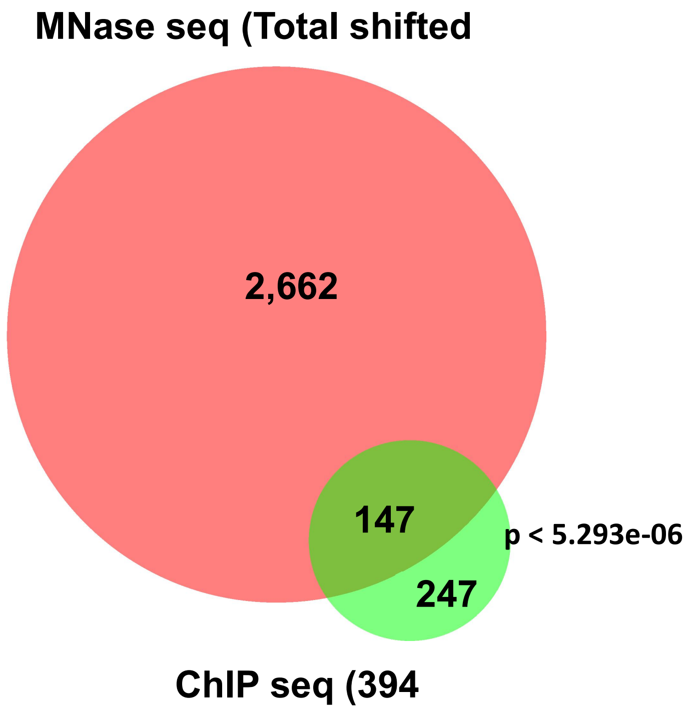

Reviewer #1 (Remarks to the Author):

The authors have provided an outstanding revision in response to my earlier comments. The new version includes high quality new data and explanations that support all of their conclusions. This is a dense, high quality piece of work. Outstanding.

Reviewer #2 (Remarks to the Author):

The authors have addressed my questions and I have no further comments.
